# Correction: Comprehensive assessment of a nationwide simulation-based course for artificial life support

**DOI:** 10.1371/journal.pone.0318273

**Published:** 2025-01-23

**Authors:** Mateusz Puslecki, Marek Dabrowski, Marcin Ligowski, Bishoy Zakhary, Ahmed S. Said, Kollengode Ramanathan, Elaine Cooley, Lukasz Puslecki, Sebastian Stefaniak, Piotr Ziemak, Ilona Kiel-Puslecka, Agata Dabrowska, Tomasz Klosiewicz, Maciej Sip, Radoslaw Zalewski, Malgorzata Ladzinska, Wojciech Mrowczynski, Piotr Ladzinski, Lidia Szlanga, Konrad Baumgart, Piotr Kupidlowski, Lukasz Szarpak, Marek Jemielity, Bartlomiej Perek

In Fig 6, the R&B Development should be R&D Development. Please see the correct Fig 6 here.

**Fig 6:**
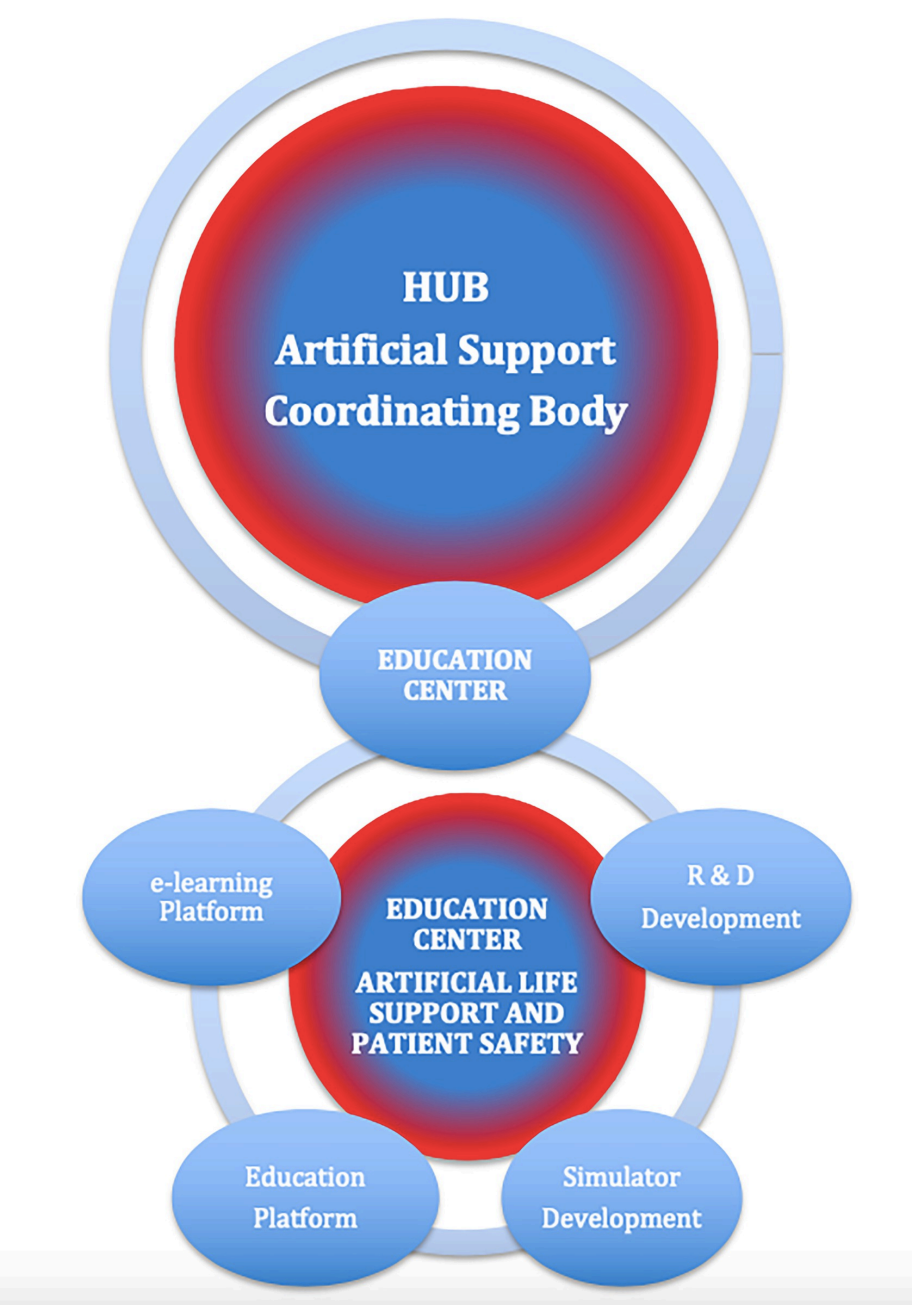
R&D development: “know-how and knowledge transfer” in education center of artificial support in “ECMO for greater Poland” innovation ecosystem.

In the Limitation subsection of Discussion, the third sentence of the first paragraph should have not been indicated. The correct first paragraph is: Up to now we can present only early results of our course and we must wait for at least some months or years to know how training in ALS impacted on clinical practice and ECMO applications. In spite of this limitation, the short-term results of developed concept in “Center of Artificial Life Support and Patient Safety” with ELSO cooperation are very promising. Moreover, having practice in ECMO applications, we are aware how important is good cooperation with many other members of medical personnel and emergency system. This course was only for physicians. Of note, during training we appeal for participants to create their own teams and to play by them role of leaders. The significant improvement in behavioral assessment, particular leader of ECMO team, gives hope for successful involvement other medical professionals.

## References

[1] Puslecki M, Dabrowski M, Ligowski M, Zakhary B, Said AS, Ramanathan K, et al. (2021) Comprehensive assessment of a nationwide simulation-based course for artificial life support. PLoS ONE 16(10): e0257162. 10.1371/journal.pone.0257162 34618829 PMC8496826

