# Supplementary material for: Comprehensive assessment of a nationwide simulation-based course for artificial life support
Source: PLoS One. 2021 Oct 7;16(10):e0257162. doi: 10.1371/journal.pone.0257162 (PMC8496826; doi:10.1371/journal.pone.0257162)
Supplement: S1 File — (PDF) [file pone.0257162.s001.pdf]

PUMS - Artificial Life Support with ECMO: Demographics & Pre-Course Self-Assessment (Dane demograficzne i samoocena uczestnika)

Please complete the following questions.

*Wymagane

Name: (Nazwisko i Imię) *

Twoja odpowiedź

Please identify your sex (Płeć) *

Wybierz

What is your age group? (Wiek?) *

Wybierz

What is your medical specialty? (Specjalność medyczna) *

Wybierz

What setting do you work in? (W jakiej instytucji pracujesz?) *

Wybierz

What is your typical patient age group? (Jaka grupa pacjentów jest leczona w Twojej instytucji?) *

- [ ] Pediatric (dzieci)
- [ ] Neonatal (noworodki)
- [ ] Adult (dorośli)

How long have you been in practice? (Ile lat wykonujesz swój zawód?) *

Wybierz

How much ECMO experience do you have? (Jakie jest Twoje doświadczenie w technikach pozaustrojowych ECMO?) *

Wybierz

How long has your hospital offered ECMO? (Od kiedy techniki pozaustrojowe ECMO są dostępne w Twojej instytucji?) *

Wybierz

What is your annual ECMO patient volume? (Liczba pacjentów leczona z wykorzystaniem technik pozaustrojowych ECMO w ciągu roku w Twojej instytucji) *

Wybierz

Which modes of ECMO are available are your hospital? (Jakiego rodzaju terapie ECMO są stosowane w Twojej instytucji?) *

Wybierz

Which ECMO pump system does your center utilize? (Które urządzenia są dostępne w Twojej instytucji?) *

Wybierz

How does your hospital handle patients requiring ECMO support? (W jakim obszarze Twoja instytucja może proponować techniki pozaustrojowe ECMO?) *

Wybierz

Dalej

Nigdy nie podawaj w Formularzach Google swoich haseł.

Ten formularz został utworzony w domenie University of Michigan. Zgłoś nadużycie

Formularze Google

PUMS - Artificial Life Support with ECMO: Demographics & Pre-Course Self-Assessment (Dane demograficzne i samoocena uczestnika)

*Wymagane

Pre-Course Self-Assessment

Please answer all questions

COGNITIVE (Umiejętności kognitywne)

1. I understand the physiological basis of extracorporeal support (Rozumiem fizjologiczne podstawy wspomagania pozaustrojowego) *

	1	2	3	4	5	
Least Confident (nie)	○	○	○	○	○	Most Confident (tak)

2. I am familiar with the components of the ECMO circuit (Znam składowe układu ECMO) *

	1	2	3	4	5	
Least Confident (nie)	○	○	○	○	○	Most Confident (tak)

3. I understand the physiologic differences between VV and VA ECMO (Rozumiem fizjologiczne różnice między VV i VA ECMO) *

	1	2	3	4	5	
Least Confident (nie)	○	○	○	○	○	Most Confident (tak)

4. I can list the indications for VA ECMO (Potrafię wymienić wskazania do VA ECMO) *

	1	2	3	4	5	
Least Confident (nie)	○	○	○	○	○	Most Confident (tak)

5. I can list the indications for VV ECMO (Potrafię wymienić wskazania do VV ECMO) *

	1	2	3	4	5	
Least Confident (nie)	○	○	○	○	○	Most Confident (tak)

6. I can list the contraindications for ECMO (Potrafię wymienić przeciwwskazania do ECMO) *

	1	2	3	4	5	
Least Confident (nie)	○	○	○	○	○	Most Confident (tak)

7. I can describe the different configurational options for VA ECMO (Potrafię opisać różne konfiguracje VA ECMO) *

	1	2	3	4	5	
Least Confident (nie)	○	○	○	○	○	Most Confident (tak)

8. I can describe the different configurational options for VV ECMO (Potrafię opisać różne konfiguracje VV ECMO) *

	1	2	3	4	5	
Least Confident (nie)	○	○	○	○	○	Most Confident (tak)

9. I understand the concept of lung rest and heart rest while on ECMO support (Rozumiem koncepcję terapii oszczędzającej płuca oraz oszczędzającej serce w trakcie wspomagania ECMO) *

	1	2	3	4	5	
Least Confident (nie)	○	○	○	○	○	Most Confident (tak)

10. I understand the process of weaning off VA ECMO (Rozumiem proces odzwyczajania od VA ECMO) *

	1	2	3	4	5	
Least Confident (nie)	○	○	○	○	○	Most Confident (tak)

11. I understand the process of weaning off VV ECMO (Rozumiem proces odzwyczajania od VV ECMO) *

	1	2	3	4	5	
Least Confident (nie)	○	○	○	○	○	Most Confident (tak)

TECHNICAL (Umiejętności techniczne)

12. I can perform a complete circuit check (Potrafię skompletować i skontrolować układ do wspomagania pozaustrojowego) *

	1	2	3	4	5	
Least Confident (nie)	○	○	○	○	○	Most Confident (tak)

13. I can prime an ECMO circuit (Potrafię wypełnić układ ECMO) *

	1	2	3	4	5	
Least Confident (nie)	○	○	○	○	○	Most Confident (tak)

14. I can safely change out an ECMO component (Potrafię bezpiecznie zmienić uszkodzone elementy układu ECMO) *

	1	2	3	4	5	
Least Confident (nie)	○	○	○	○	○	Most Confident (tak)

15. I can remove air from an ECMO circuit (Potrafię usunąć powietrze z układu ECMO) *

	1	2	3	4	5	
Least Confident (nie)	○	○	○	○	○	Most Confident (tak)

16. I can troubleshoot oxygenator failure (Potrafię rozwiązać problemy uszkodzonego oksygenatora) *

	1	2	3	4	5	
Least Confident (nie)	○	○	○	○	○	Most Confident (tak)

17. I can manage pump failure on ECMO (Potrafię rozwiązać problem uszkodzonego napędu ECMO) *

	1	2	3	4	5	
Least Confident (nie)	○	○	○	○	○	Most Confident (tak)

BEHAVIORAL (Umiejętności behawioralne)

18. I feel confident in my role as a member of the ECMO team (Czuję się pewnie jako członek ECMO zespołu) *

	1	2	3	4	5	
Least Confident (nie)	○	○	○	○	○	Most Confident (tak)

19. I can lead a team caring for a VV ECMO patient (Mogę być liderem zespołu w trakcie prowadzenia VV ECMO) *

	1	2	3	4	5	
Least Confident (nie)	○	○	○	○	○	Most Confident (tak)

20. I can lead a team caring for a VA ECMO patient (Mogę być liderem zespołu w trakcie prowadzenia VA ECMO) *

	1	2	3	4	5	
Least Confident (nie)	○	○	○	○	○	Most Confident (tak)

21. I can emergently come off ECMO and support a patient when needed (Potrafię zabezpieczyć funkcje życiowe pacjenta w razie pilnej potrzeby wyjścia z ECMO) *

	1	2	3	4	5	
Least Confident (nie)	○	○	○	○	○	Most Confident (tak)

Wstecz   Prześlij

Nigdy nie podawaj w Formularzach Google swoich haseł.

Ten formularz został utworzony w domenie University of Michigan. Zgłoś nadużycie

Formularze Google
