# Supplementary material for: Comprehensive assessment of a nationwide simulation-based course for artificial life support
Source: PLoS One. 2021 Oct 7;16(10):e0257162. doi: 10.1371/journal.pone.0257162 (PMC8496826; doi:10.1371/journal.pone.0257162)
Supplement: S2 File — (DOCX) [file pone.0257162.s002.docx]

**Supplementary material 2.** Scope of knowledge, objectives and skills.

|  | SCOPE OF KNOWLEDGE: |
| --- | --- |
| 1 | „Rescue chain of survival” - how to build and modify it to increase survival rates; |
| 2 | High-quality basic and advanced resuscitation procedures; the use of automatic mechanical chest compression; |
| 3 | Patients and staff safety during perfusion support using ECMO; |
| 4 | Introduction to ECMO technology: history, current state of knowledge; risk assessment scales used in extracorporeal techniques; membrane gas exchange physics and physiology; oxygen content, delivery and consumption; shunt physiology |
| 5 | Pathophysiology of diseases treated with ECLS/ECMO extracorporeal support (Persistent Pulmonary Hypertension; Meconium Aspiration Syndrome; Respiratory Distress Syndrome; Congenital Diaphragmatic Hernia; Sepsis/pneumonia; Post-operative congenital heart disease/heart transplantation; Drugs Intoxication; Cardiomyopathy/myocarditis; ARDS; Aspiration pneumonia; Pulmonary embolism); |
| 6 | Implementation criteria for ECMO in accordance with current standards (Recommendations and Guidelines of the ECMO Vascular Venous Therapy Team, appointed by the National Consultant for Anesthesiology and Intensive Care in Poland, ELSO); patient selection, inclusion and exclusion criteria; contraindications; the type of support in difference protocols (VV, VA, ECPR, Hypothermia, DCD) |
| 7 | Pathological changes in physiology of a patient undergoing VA /VV-ECMO; |
| 8 | Devices for extracorporeal perfusion therapy and all components of the system, including differences for pediatric patients and individual therapeutic protocols; |
| 9 | Manual for the ECLS/ECMO system and intervention protocols for emergency situations; |
| 10 | Principles in extracorporeal perfusion support; |
| 11 | Therapy of basic diseases resistant to conventional therapy in patients assisted extracorporeally; |
| 12 | Treatment with blood products and anti-coagulation during ECMO therapy; |
| 13 | Monitoring of the ECMO support therapy; |
| 14 | Methods for monitoring the circulatory system of a patient undergoing VA/VV-ECMO; |
| 15 | Medical complications and life-threatening conditions: bleeding to CNS/strokes; renal failure; coagulopathy; hypertension/hypotension; hypovolemia; cardiac arrest; |
| 16 | ECMO weaning protocols; |
| 17 | Criteria for support termination; |
| 18 | Ethical and social issues: consent to ECMO; support for parents/families; communication with the patient and his family; |
| 19 | Evaluation of long-term outcomes of ECMO therapy application; |
| 20 | The role of the interdisciplinary ECMO team; perfusion, nursing and rehabilitation care; |
| 21 | "ECMO Team" and "Mobile ECMO Team". |
|  | OBJECTIVES AND SKILLS: |
| 1 | Organization of the ECMO team, communication in a team, cooperation with other team members participating in the therapy (perfusionist, nurse) in the field of assessment of the patient's clinical status and related therapeutic decisions; |
| 2 | High quality cardiopulmonary resuscitation. The use of automatic mechanical chest compression in the ALS algorithm; ECMO-DCD and ECMO-CPR / ECPR protocols; |
| 3 | Operating an ultrasound with a linear and sector probe; |
| 4 | Evaluation and selection of vascular access to peripheral percutaneous cannulation - normal anatomy, including anatomical variability of the vessels - variants and anatomical variations - methods of managing existing peripheral vascular pathology; |
| 5 | Percutaneous cannulation under ultrasound guidance (assessment of vessel size and proper cannula selection); correct vessel puncture by the Seldinger method under ultrasound control by the operator; vascular shunt for peripheral perfusion; Surgical technique and its variants; |
| 6 | Ultrasound examination (sectoral and transesophageal probe) of the arterial and venous cannulas in the inferior and superior vena cava and the position of the Avalon cannula in the right atrium; |
| 7 | Preparation of the ECMO circuit; priming of the extracorporeal system, venting the system and cannulas; connection to ECMO in different variants of the drainage system: VV, VA, VAV; |
| 8 | Daily clinical evaluation of a patient on ECMO, care, assessment of nutritional status, neurological assessment, pain control and the need for sedation, monitoring of the respiratory/circulatory system functions, psycho-social aspects/information for the patient's family; |
| 9 | Daily assessment of the ECMO extracorporeal system: application of aseptic rules, determination of gas and blood flow through the pump, monitoring of perfusion parameters, evaluation of anticoagulation efficacy, system tests, prevention of aeration, switching to emergency drive in the event of damage to the ECMO pump; |
| 10 | Hemofiltration circuit connection/disconnection to ECMO circuit; |
| 11 | Interpretation and ECMO alarms interventions; |
| 12 | Treatment of complications and life-threatening conditions during extracorporeal support; |
| 13 | Mechanical complications/hardware failures: system damage, air in the system, cavitation, thrombi, clotting of the system, pump stop, cannula migration; |
| 14 | Non-standard interventions during ECMO: surgical procedures in patients undergoing ECMO therapy, bleeding, cardiac catheterization, diagnostic imaging tests (CT, angiography); intra and interhospital transportation; |
| 15 | Complications accompanying vascular cannulation - the most common complications of percutaneous cannulation, technical and diagnostic errors; management to avoid damage to vessels and nerves; the role of the interdisciplinary team in the treatment of complications; |
| 16 | Recovery on ECMO; ECMO weaning; pulmonary and/or myocardial regeneration determinants, techniques for reducing perfusate flow on the blood pump and gases, methods of assessing the function of the supported organ; |
| 17 | Decannulation: surgical techniques, additional surgeries, ultrasound assessment; |
| 18 | Additional support forms – rotation and centrifugal pumps; |
| 19 | Techniques of LV uploading; |
| 20 | Medical documentation during ECMO support - card of perfusion. |
