# Supplementary material for: Comprehensive assessment of a nationwide simulation-based course for artificial life support
Source: PLoS One. 2021 Oct 7;16(10):e0257162. doi: 10.1371/journal.pone.0257162 (PMC8496826; doi:10.1371/journal.pone.0257162)
Supplement: S3 File — (DOCX) [file pone.0257162.s003.docx]

**Supplementary material 3.** 3-day ALS course program.

| Day 1: 14:00-19:00 - **ALS – ARTIFICIAL LIFE SUPPORT** | |  | |
| --- | --- | --- | --- |
| **Topics:**   - Introduction to ALS - Artificial Life Support. - Indications and contraindications for ECMO support. - Basics of therapy, components, preparation, operation, and monitoring of the system. - Types of cannulation and methods of initiating the therapy. - Differences in the therapy of patients with extracorporeal support. - Weaning from ECMO.   Theoretical background of:   - High quality CPR - ECMO – basics - ECMO – elements and maintenance of the circuit - ECMO – system monitoring - Techniques and types of cannulation - Basics of initiating the therapy - Types of extracorporeal therapy with pediatric and adult patients - Indications, contraindications, and differences between VV and VA ECMO   - ECMO VV   - ECMO VA   - ECMO CPR   - ECMO HYPOTHERMIA   - ECMO DCD - ECMO recovery, weaning, and therapy termination | |  | |
| Day 2: 9:00-14:00 - **WORKSHOPS – „TIPS & TRICKS” (parallel in 3 subgroups of 4 candidates)** | |  | |
| **Topics:**   - - ECMO VV   - ECMO VA   - ECMO CPR   - ECMO HIPOTERMIA   - ECMO DCD - Imaging techniques   - Ultrasound imaging in ECMO   - Echocardiographic imaging in ECMO - Cannulation techniques – percutaneous/surgery   - ECMO – VV cannulation   - ECMO – VA cannulation - Preparation of ECMO devices available on the Polish market   - Preparation and operation of perfusion devices   - Priming devices for extracorporeal perfusion - High quality CPR – quality improvement - Mechanical automatic chest compression in the ALS protocol - Intra- and interhospital transportation - Communication in team and prebriefing | |  | |
| Day 2: 15:00-19:00 and Day 3: 9:00-13:00 - **SIMULATION TRAINING** | | |  |
| **Exercises based on immersive medical simulation.** Practical implementation of protocols including critical moments of therapy. Proper communication within the therapeutic team, patient, and the family. | | |  |
| **Training**  **scenarios** | Simulation scenarios using a patient simulator and ECMO simulator based on the equipment used clinically. List of scenarios (Table 2). | |  |
| Day 3: 13:30-15:00 - **EXAMINATION** | | | |
| TEST – 15 multiple-choice questions | | | |
| **Practical examination** | Simulation scenarios using a patient simulator – random selection of a scenario | | |
