# Supplementary material for: Comprehensive assessment of a nationwide simulation-based course for artificial life support
Source: PLoS One. 2021 Oct 7;16(10):e0257162. doi: 10.1371/journal.pone.0257162 (PMC8496826; doi:10.1371/journal.pone.0257162)
Supplement: S4 File — (DOCX) [file pone.0257162.s004.docx]

**Supplementary material 4.** List of immersive scenarios for ECMO therapy and corresponding learning outcomes.

|  | **SCENARIO** | **LEARNING OUTCOMES** |
| --- | --- | --- |
| 1 | Accidental hypovolemia | Ability to diagnose hypovolemia symptoms during VV ECMO treatment and solve problem |
| 2 | Circuit aeration | Ability to diagnose ECMO circuit aeration and safe de-airing procedure within 10 minutes while maintaining patient ventilation |
| 3 | Accidental circuit disconnection | Ability to diagnose the ECMO circuit disconnection, minimize bleeding, maintain patient ventilation, and re-initiate the ECMO run following cannula reconnection within 3-5 minutes |
| 4 | System clotting | Ability to recognize symptoms of clot formation in the circuit and to change a circuit within 15 minutes |
| 5 | Gas supply line obstruction | Ability to recognize symptoms of gas line obstruction and safely troubleshoot issue within 5 minutes while performing chest compressions and bag-valve-mask ventilation |
| 6 | No power supply - manual cranking | Ability to recognize the lack of power supply and change to manual cranking |
| 7 | Emergency ECMO cannulation | Ability to perform femoral VA cannulation on a patient – undergoing resuscitation with an external chest compressions device - ECPR |
| 8 | Hemofiltration connection | Ability to connect the hemofiltration circuit via drains to the ECMO system |
| 9 | Intrahospital transportation | Ability to safely perform an ECMO patient intrahospital transportation i.e. using a lift, going to CT scan, etc. |
| 10 | Interhospital transportation | Ability to demonstrate how to prepare for and safely perform an interhospital transportation with an ECMO patient |
