# Supplementary material for: Comprehensive assessment of a nationwide simulation-based course for artificial life support
Source: PLoS One. 2021 Oct 7;16(10):e0257162. doi: 10.1371/journal.pone.0257162 (PMC8496826; doi:10.1371/journal.pone.0257162)
Supplement: S5 File — (PDF) [file pone.0257162.s005.pdf]

PUMS Artificial Life Support with ECMO: Post-Self Assessment & Course Evaluation (Ocena zdobytych umiejętności i ocena kursu)

Please answer all questions

*Wymagane

Name (Nazwisko i Imię) *

Twoja odpowiedź

COGNITIVE (Umiejętności kognitywne)

1. I understand the physiological basis of extracorporeal support (Rozumiem fizjologiczne podstawy wspomagania pozaustrojowego) *

	1	2	3	4	5	
Least Confident (nie)	○	○	○	○	○	Most Confident (tak)

2. I am familiar with the components of the ECMO circuit (Znam składowe układu ECMO) *

	1	2	3	4	5	
Least Confident (nie)	○	○	○	○	○	Most Confident (tak)

3. I understand the physiologic differences between VV and VA ECMO (Rozumiem fizjologiczne różnice pomiędzy VV i VA ECMO) *

	1	2	3	4	5	
Least Confident (nie)	○	○	○	○	○	Most Confident (tak)

4. I can list the indications for VA ECMO (Potrafię wymienić wskazania do VA ECMO) *

	1	2	3	4	5	
Least Confident (nie)	○	○	○	○	○	Most Confident (tak)

5. I can list the indications for VV ECMO (Potrafię wymienić wskazania do VV ECMO) *

	1	2	3	4	5	
Least Confident (nie)	○	○	○	○	○	Most Confident (tak)

6. I can list the contraindications for ECMO (Potrafię wymienić przeciwwskazania do ECMO) *

	1	2	3	4	5	
Least Confident (nie)	○	○	○	○	○	Most Confident (tak)

7. I can describe the different configurational options for VA ECMO (Potrafię opisać różne konfiguracje VA ECMO) *

	1	2	3	4	5	
Least Confident (nie)	○	○	○	○	○	Most Confident (tak)

8. I can describe the configurational options for VV ECMO (Potrafię opisać różne konfiguracje VV ECMO) *

	1	2	3	4	5	
Least Confident (nie)	○	○	○	○	○	Most Confident (tak)

9. I understand the concept of lung rest and heart rest while on ECMO support (Rozumiem koncepcję terapii oszczędzającej płuca oraz oszczędzającej serce w trakcie wspomagania ECMO) *

	1	2	3	4	5	
Least Confident (nie)	○	○	○	○	○	Most Confident (tak)

10. I understand the process of weaning off VA ECMO (Rozumiem proces odzwyczajania od VA ECMO) *

	1	2	3	4	5	
Least Confident (nie)	○	○	○	○	○	Most Confident (tak)

11. I understand the process of weaning off VV ECMO (Rozumiem proces odzwyczajania od VV ECMO) *

	1	2	3	4	5	
Least Confident (nie)	○	○	○	○	○	Most Confident (tak)

TECHNICAL (Umiejętności techniczne)

12. I can perform a complete circuit check (Potrafię skompletować i skontrolować układ do wspomagania pozaustrojowego) *

	1	2	3	4	5	
Least Confident (nie)	○	○	○	○	○	Most Confident (tak)

13. I can prime an ECMO circuit (Potrafię wypełnić układ ECMO) *

	1	2	3	4	5	
Least Confident (nie)	○	○	○	○	○	Most Confident (tak)

14. I can safely change out an ECMO component (Potrafię bezpiecznie zmienić uszkodzone elementy układu ECMO) *

	1	2	3	4	5	
Least Confident (nie)	○	○	○	○	○	Most Confident (tak)

15. I can remove air from an ECMO circuit (Potrafię usunąć powietrze z układu ECMO) *

	1	2	3	4	5	
Least Confident (nie)	○	○	○	○	○	Most Confident (tak)

16. I can troubleshoot oxygenator failure (Potrafię rozwiązać problemy uszkodzonego oksygenatora) *

	1	2	3	4	5	
Least Confident (nie)	○	○	○	○	○	Most Confident (tak)

17. I can manage acute pump failure on ECMO (Potrafię rozwiązać problem uszkodzonego napędu ECMO) *

	1	2	3	4	5	
Least Confident (nie)	○	○	○	○	○	Most Confident (tak)

BEHAVIORAL (Umiejętności behawioralne)

18. I feel confident in my role as a member of the ECMO team (Czuję się pewnie jako członek ECMO zespołu) *

	1	2	3	4	5	
Least Confident (nie)	○	○	○	○	○	Most Confident (tak)

19. I can lead a team caring for a VV ECMO patient (Mogę być liderem zespołu w trakcie prowadzenia VV ECMO) *

	1	2	3	4	5	
Least Confident (nie)	○	○	○	○	○	Most Confident (tak)

20. I can lead a team caring for a VA ECMO patient (Mogę być liderem zespołu w trakcie prowadzenia VA ECMO) *

	1	2	3	4	5	
Least Confident (nie)	○	○	○	○	○	Most Confident (tak)

21. I can emergently come off ECMO and support a patient when needed (Potrafię zabezpieczyć funkcje życiowe pacjenta w razie pilnej potrzeby wyjścia z ECMO) *

	1	2	3	4	5	
Least Confident (nie)	○	○	○	○	○	Most Confident (tak)

COURSE EVALUATION (Ocena kursu)

DIDACTIC (Dydaktyka)

1. The lectures were well prepared (Przygotowanie wykładów) *

	1	2	3	4	5	
POOR (słabo)	○	○	○	○	○	EXCELLENT (doskonale)

2. The lectures were relevant to my clinical practice (Wykłady były istotne dla mojej praktyki klinicznej) *

	1	2	3	4	5	
POOR (słabo)	○	○	○	○	○	EXCELLENT (doskonale)

3. The content increased my knowledge of ECMO management (Przekazane informacje wzbogaciły moją wiedzę o technikach pozaustrojowych) *

	1	2	3	4	5	
POOR (słabo)	○	○	○	○	○	EXCELLENT (doskonale)

4. The presenters were knowledgeable in the content (Profesjonalizm i przygotowanie wykładowców) *

	1	2	3	4	5	
POOR (słabo)	○	○	○	○	○	EXCELLENT (doskonale)

SIMULATION (Symulacje)

5. The simulations were well organized (Organizacja zajęć symulacyjnych) *

	1	2	3	4	5	
POOR (słaba)	○	○	○	○	○	EXCELLENT (doskonała)

6. The simulations were clinically appropriate (Wartość kliniczna scenariuszy symulacyjnych) *

	1	2	3	4	5	
POOR (słaba)	○	○	○	○	○	EXCELLENT (doskonała)

7. The simulations were realistic (Realizm scenariuszy symulacyjnych) *

	1	2	3	4	5	
POOR (słaby)	○	○	○	○	○	EXCELLENT (doskonały)

8. The simulation debriefing was effective (Wartość komentarzy w debriefingu) *

	1	2	3	4	5	
POOR (słaba)	○	○	○	○	○	EXCELLENT (doskonała)

9. The simulations have improved my communication skills (Scenariusze symulacyjne wzmocniły moje umiejętności komunikacyjne) *

	1	2	3	4	5	
POOR (słabo)	○	○	○	○	○	EXCELLENT (doskonale)

10. The simulations enhanced my knowledge of ECMO (Scenariusze symulacyjne wzmocniły moją wiedzę o ECMO)

	1	2	3	4	5	
POOR (słabo)	○	○	○	○	○	EXCELLENT (doskonale)

FACULTY (Ocena zajęć)

9. The faculty were knowledgeable (Wartość merytoryczna) *

	1	2	3	4	5	
POOR (słaba)	○	○	○	○	○	EXCELLENT (doskonała)

10. The faculty were engaged (Atrakcyjność zajęć) *

	1	2	3	4	5	
POOR (słaba)	○	○	○	○	○	EXCELLENT (doskonała)

11. The faculty discussions were clinically oriented (Merytoryczność dyskusji) *

	1	2	3	4	5	
POOR (słaba)	○	○	○	○	○	EXCELLENT (doskonała)

FACILITIES (Udogodnienia)

12. Facilities were clean and easily accessible (Warunki lokalowe) *

	1	2	3	4	5	
POOR (słabe)	○	○	○	○	○	EXCELLENT (doskonałe)

13. Audiovisuals were appropriate for the presentations (Systemy i techniki audiowizualne) *

	1	2	3	4	5	
POOR (słabe)	○	○	○	○	○	EXCELLENT (doskonałe)

14. Facilities and site were easy to navigate with clear directions (Otoczenie i pomieszczenia były prawidłowo oznaczone) *

	1	2	3	4	5	
POOR (słabo)	○	○	○	○	○	EXCELLENT (doskonale)

LOGISTICS (Logistyka)

15. I am satisfied with the registration process (Proces rejestracji był satysfakcjonujący) *

	1	2	3	4	5	
POOR (słabo)	○	○	○	○	○	EXCELLENT (doskonale)

16. I am satisfied with the program organization (Organizacja i realizacja programu) *

	1	2	3	4	5	
POOR (słaba)	○	○	○	○	○	EXCELLENT (doskonała)

17. The program was valuable to me (Wartość programu szkoleniowego) *

	1	2	3	4	5	
POOR (słaby)	○	○	○	○	○	EXCELLENT (doskonały)

18. I would recommend this course to others (Czy zarekomendujesz ten kurs innym jako kurs) *

	1	2	3	4	5	
POOR (słaby)	○	○	○	○	○	EXCELLENT (doskonały)

19. I would take this course again (Czy wziętabyś/wziąłbyś ponownie udział w takim kursie) *

	1	2	3	4	5	
POOR (zdecydowanie nie)	○	○	○	○	○	EXCELLENT (zdecydowanie tak)

GENERAL IMPRESSIONS (Ogólna ocena i odbiór kursu)

20. How useful for your professional activity did you find the course (Czy kurs był przydatny dla Twojej przyszłej profesjonalnej działalności) *

	1	2	3	4	5	
POOR (zdecydowanie nie)	○	○	○	○	○	EXCELLENT (zdecydowanie tak)

21. What was your overall impression of this course? (Jakie są Twoje wrażenia po kursie?) *

	1	2	3	4	5	
POOR (słabe)	○	○	○	○	○	EXCELLENT (doskonałe)

22. Did the course fulfill your educational goals and expected learning outcomes (Czy kurs zaspokoił Twoje oczekiwania edukacyjne?) *

Wybierz

23. Was the presented information well balanced and consistently supported by a valid scientific evidence base? (Czy zaprezentowane informacje były merytoryczne i przekazane w przystępnej formie?) *

Wybierz

24. Was there adequate time available for discussions, questions & answers, and learned engagement? (Czy czas na dyskusję, pytania i odpowiedzi był wystarczający?) *

Wybierz

25. Will the information you learned be implemented in your practice? (Czy przekazane informacje zostaną przez Ciebie wykorzystane w praktyce?) *

Wybierz

26. Did the faculty members provide their potential conflicts of interest declaration with the sponsor(s) as a second slide of their presentation? (Czy wykładowcy przedstawili potencjalne konflikty interesów lub powiązania z firmami i sponsorami na drugim przezroczu prezentacji?) *

Wybierz

27. Do you agree that the information was overall free of commercial and other bias? (Czy przekazane informacje byłu wolne od komercyjnych powiązań z firmami i sponsorami?) *

Wybierz

28. If there was bias, please list here: (Jeśli wystąpiły niedogodności, proszę wymienić tutaj.)

Twoja odpowiedź

29. What were the motivations for participating in the Course? (Jakie były Twoje motywacje do uczestnictwa w Kursie?) *

Wybierz

30. If you responded "other" to question 29, please provide reason here(Jeśli wybrałeś "inny" dla pytania 29. podaj proszę powód):

Twoja odpowiedź

Please provide any additional comments here (Miejsce na dodatkowe uwagi i komentarze)

Twoja odpowiedź

Pobierz link

Nigdy nie podawaj w Formularzach Google swoich haseł.

Ten formularz został utworzony w domenie University of Michigan. Zgłoś nadużycie

Formularze Google
