# Supplementary material for: Comprehensive assessment of a nationwide simulation-based course for artificial life support
Source: PLoS One. 2021 Oct 7;16(10):e0257162. doi: 10.1371/journal.pone.0257162 (PMC8496826; doi:10.1371/journal.pone.0257162)
Supplement: Attachment — Submitted filename: PONE-D-21-14816_reviewer - comments 6 July 2021.pdf [file pone.0257162.s007.pdf]

PLOS ONE

Efficacy of nationwide simulation based Artificial Life Support training during the COVID-19 pandemic year

--Manuscript Draft--

**Manuscript Number:**	PONE-D-21-14816
**Article Type:**	Research Article
**Full Title:**	Efficacy of nationwide simulation based Artificial Life Support training during the COVID-19 pandemic year
**Short Title:**	Nationwide simulation based Artificial Life Support training
**Corresponding Author:**	Mateusz Puślecki<br>Poznan University of Medical Sciences<br>Poznań, POLAND
**Keywords:**	ECMO; simulation, education, critical care, artificial life support, knowledge transfer, innovation cooperation, R&D alliances, business-academia cooperation
**Abstract:**	BACKGROUND<br><br>Successful implementation of the medical technologies applied in life-threatening conditions, including extracorporeal membrane oxygenation (ECMO) requires appropriate preparation and training of medical personnel. The pandemic has accelerated the creation of new ECMO centers and has highlighted continuous training in adapting to new pandemic standards. To reach high standards of patients' care, we created the first of its kind, National Education Centre for Artificial Life Support (NEC-ALS) in 40 million inhabitants' country in the Central and Eastern Europe (CEE). The role of the Center is to test and promote the novel or commonly used procedures as well as to develop staff skills on management of patients needing ECMO.<br><br>METHOD<br><br>In 2020, nine approved and endorsed by ELSO courses of "Artificial Life Support with ECMO" were organized. Physicians participated in the three-day high-fidelity simulation-based training that was adapted to abide by the social distancing norms of the COVID-19 pandemic. Knowledge as well as crucial cognitive, behavioral and technical aspects (on a 5-point Likert scale) of management on ECMO were assessed before and after course completion. Moreover, the results of training in mechanical chest compression were also evaluated.<br><br>RESULTS<br><br>There were 115 participants (60% men) who were young (31-40 years). Majority of them (63%) were anesthesiologists or intensivists with more than 5-year clinical experience, but 54% had no previous ECMO experience. There was significant improvement in all cognitive, behavioral, and technical self-assessments after the course. Among aspects of management with ECMO that all increased significantly following the course, the most pronounced was related to the technical one (from approximately 1.0 to more 4.0 points). Knowledge scores significantly increased post-course from 11.4 ± SD to 13 ± SD (out of 15 points). The quality of manual chest compression relatively poor before course improved significantly after training.<br><br>CONCLUSIONS<br><br>Our course confirmed that simulation as an educational approach is invaluable not only in training and testing of novel or commonly used procedures, skills upgrading, but also in practicing very rare cases. The implementation of the education program during COVID-19 pandemic may be helpful in founding specialized Advanced Life Support centers and teams including mobile ones. The dedicated R&D Innovation Ecosystem established in the "ECMO for Greater Poland" program, with developed National Education Center can play a crucial role in the knowledge and know-how transfer but future research is needed.

Powered by Editorial Manager® and ProduXion Manager® from Aries Systems Corporation

Order of Authors:	Mateusz Puślecki
	Marek Dabrowski
	Marcin Ligowski
	Bishoy Zakhary
	Ahmed S. Said
	Kollengode Ramanathan
	Elaine Cooley
	Lukasz Puslecki
	Sebastian Stefaniak
	Piotr Ziemak
	Ilona Kiel-Puslecka
	Agata Dąbrowska
	Tomasz Kłosiewicz
	Maciej Sip
	Radoslaw Zalewski
	Malgorzata Ladzinska
	Wojciech Mrowczyński
	Piotr Ladziński
	Lidia Szlanga
	Konrad Baumgart
	Piotr Kupidlowski
	Lukasz Szarpak
	Marek Jemielity
	Bartlomiej Perek
Additional Information:	
Question	Response
**Financial Disclosure**<br><br>Enter a financial disclosure statement that describes the sources of funding for the work included in this submission. Review the submission guidelines for detailed requirements. View published research articles from *PLOS ONE* for specific examples.<br><br>This statement is required for submission and **will appear in the published article** if the submission is accepted. Please make sure it is accurate.	Yes. The project was awarded funding from a POWER competitive national grant (POWR.05.04.00-IP.05-00-006/18) by the Polish Ministry of Health for a total of 2,750,000 USD (PLN 10,974,708.60). Main reason was to develop a course about "Artificial Life Support with ECMO" offered to 264 physicians from Poland implemented in 2019-2021 at the Poznan University of Medical Sciences (PUMS).

Powered by Editorial Manager® and ProduXion Manager® from Aries Systems Corporation

**Unfunded studies**<br>Enter: *The author(s) received no specific funding for this work.*<br><br>**Funded studies**<br>Enter a statement with the following details:<br>• Initials of the authors who received each award<br>• Grant numbers awarded to each author<br>• The full name of each funder<br>• URL of each funder website<br>• Did the sponsors or funders play any role in the study design, data collection and analysis, decision to publish, or preparation of the manuscript?<br>• **NO** - Include this sentence at the end of your statement: *The funders had no role in study design, data collection and analysis, decision to publish, or preparation of the manuscript.*<br>• **YES** - Specify the role(s) played.<br><br>* typeset	
**Competing Interests**<br><br>Use the instructions below to enter a competing interest statement for this submission. On behalf of all authors, disclose any competing interests that could be perceived to bias this work—acknowledging all financial support and any other relevant financial or non-financial competing interests.<br><br>This statement is required for submission and **will appear in the published article** if the submission is accepted. Please make sure it is accurate and that any funding sources listed in your Funding Information later in the submission form are also declared in your Financial Disclosure statement.<br><br>View published research articles from *PLOS ONE* for specific examples.	The authors have declared that no competing interests exist.

Powered by Editorial Manager® and ProduXion Manager® from Aries Systems Corporation

**NO authors have competing interests**<br><br>Enter: *The authors have declared that no competing interests exist.*<br><br>**Authors with competing interests**<br><br>Enter competing interest details beginning with this statement:<br><br>*I have read the journal's policy and the authors of this manuscript have the following competing interests: [insert competing interests here]*<br><br>* typeset	
**Ethics Statement**<br><br>Enter an ethics statement for this submission. This statement is required if the study involved:<br><br>• Human participants<br>• Human specimens or tissue<br>• Vertebrate animals or cephalopods<br>• Vertebrate embryos or tissues<br>• Field research<br><br>Write "N/A" if the submission does not require an ethics statement.<br><br>General guidance is provided below. Consult the submission guidelines for detailed instructions. **Make sure that all information entered here is included in the Methods section of the manuscript.**	According to the rules of Local Bioethical Committee of Poznan University of Medical Sciences the Statement of Ethics Approval is not required.

Powered by Editorial Manager® and ProduXion Manager® from Aries Systems Corporation

**Format for specific study types**<br><br>**Human Subject Research (involving human participants and/or tissue)**<br>• Give the name of the institutional review board or ethics committee that approved the study<br>• Include the approval number and/or a statement indicating approval of this research<br>• Indicate the form of consent obtained (written/oral) or the reason that consent was not obtained (e.g. the data were analyzed anonymously)<br><br>**Animal Research (involving vertebrate animals, embryos or tissues)**<br>• Provide the name of the Institutional Animal Care and Use Committee (IACUC) or other relevant ethics board that reviewed the study protocol, and indicate whether they approved this research or granted a formal waiver of ethical approval<br>• Include an approval number if one was obtained<br>• If the study involved *non-human primates*, add *additional details* about animal welfare and steps taken to ameliorate suffering<br>• If anesthesia, euthanasia, or any kind of animal sacrifice is part of the study, include briefly which substances and/or methods were applied<br><br>**Field Research**<br><br>Include the following details if this study involves the collection of plant, animal, or other materials from a natural setting:<br>• Field permit number<br>• Name of the institution or relevant body that granted permission	
**Data Availability**<br><br>Authors are required to make all data underlying the findings described fully available, without restriction, and from the time of publication. PLOS allows rare exceptions to address legal and ethical concerns. See the PLOS Data Policy and FAQ for detailed information.	Yes - all data are fully available without restriction

A Data Availability Statement describing where the data can be found is required at submission. Your answers to this question constitute the Data Availability Statement and **will be published in the article**, if accepted.<br><br>**Important:** Stating 'data available on request from the author' is not sufficient. If your data are only available upon request, select 'No' for the first question and explain your exceptional situation in the text box.<br><br>Do the authors confirm that all data underlying the findings described in their manuscript are fully available without restriction?	
**Describe where the data may be found in full sentences. If you are copying our sample text, replace any instances of XXX with the appropriate details.**<br><br>• If the data are **held or will be held in a public repository**, include URLs, accession numbers or DOIs. If this information will only be available after acceptance, indicate this by ticking the box below. For example: *All XXX files are available from the XXX database (accession number(s) XXX, XXX.)*.<br>• If the data are all contained **within the manuscript and/or Supporting Information files**, enter the following: *All relevant data are within the manuscript and its Supporting Information files.*<br>• If neither of these applies but you are able to provide **details of access elsewhere**, with or without limitations, please do so. For example:<br><br>*Data cannot be shared publicly because of [XXX]. Data are available from the XXX Institutional Data Access / Ethics Committee (contact via XXX) for researchers who meet the criteria for access to confidential data.*<br><br>*The data underlying the results presented in the study are available from (include the name of the third party*	N/A

and contact information or URL).<br>• This text is appropriate if the data are owned by a third party and authors do not have permission to share the data.<br><br>* typeset	
Additional data availability information:	

Powered by Editorial Manager® and ProduXion Manager® from Aries Systems Corporation

Manuscript Click here to access/download;Manuscript;APEPH.docx

Efficacy of nationwide simulation based Artificial Life Support training during the COVID-19 pandemic year

Short title: **Nationwide simulation based Artificial Life Support training**

Mateusz Puslecki[1,2,3], Marek Dabrowski[3,4], Marcin Ligowski[2], Bishoy Zakhary[5], Ahmed S. Said[6], Kollengode Ramanathan[7,8,9], Elaine Cooley[10], Lukasz Puslecki[11]; Sebastian Stefaniak[2], Piotr Ziemak[12]; Ilona Kiel-Puslecka[12]; Agata Dąbrowska[1,3], Tomasz Kłosiewicz[1], Maciej Sip[1], Radoslaw Zalewski[1], Malgorzata Ladzinska[2], Wojciech Mrowczyński[31], Piotr Ladziński[13], Lidia Szlanga[3], Konrad Baumgart[2], Piotr Kupidlowski[3], Lukasz Szarpak[3,14,15]; Marek Jemielity[2], Bartlomiej Perek[2]

1. Department of Medical Rescue, Chair of Emergency Medicine, Poznan University of Medical Sciences, Collegium Adama Wrzoska Rokietnicka Street 7, Poznan 60-806, Poland
2. Department of Cardiac Surgery and Transplantology, Chair of Cardiac and Thoracic Surgery, Poznan University of Medical Sciences, Dluga Street 1/2, Poznan 61–848, Poland
3. Polish Society of Medical Simulation, Slupca, Poland
4. Chair and Department of Medical Education, Poznan University of Medical Sciences, Collegium Adama Wrzoska Rokietnicka Street 7, Poznan 60-806, Poland
5. Division of Pulmonary and Critical Care Medicine, Oregon Health and Science University, 3181 SW Sam Jackson Park Rd, Portland, Portland, OR, USA
6. Division of Pediatric Critical Care Medicine, Washington University School of Medicine in St Louis and St Louis Children's Hospital, 660 S Euclid Ave, St. Louis, Missouri, USA
7. Cardiothoracic Intensive Care Unit, National University Hospital, National University of Singapore, 5 Lower Kent Ridge Rd, Singapur 119074 Singapore
8. Yong Loo Lin School of Medicine, National University of Singapore, Kent Ridge Road Singapore 119228 Singapore
9. Bond University, Robina, QLD 14 University Dr, Robina QLD 4226, Australia;
10. Extracorporeal Life Support Organization, 3001 Miller Rd 11 Ann Arbor, Michigan 48103; USA
11. Department of International Management, Poznan University of Economics and Business, Poznan, Poland, 10 Niepodleglosci Av., 61-875 Poznan, Poland
12. Center of Medical Simulation, Poznan University of Medical Sciences, Poznan, Poland
13. Department of Pediatric Cardiac Surgery, Poznan University of Medical Sciences, Poznan, Poland
14. Sklodowska-Curie Medical Academy, Warsaw, Poland
15. Polish Society of Disaster Medicine, Warsaw, Poland

Correspondence should be addressed to Mateusz Puslecki; mateuszpuslecki@o2.pl

Mateusz Puslecki MD, PhD
Department of Cardiac Surgery and Transplantology
Dluga Street ½
61-848 Poznan
Phone: 0048 618549233
Mail: mateuszpuslecki@o2.pl
ORCID iD: 0000-0003-0015-2808

Author Contributions: Course program construction: MP, MD, ML, LS, BP, MJ; ELSO endorsement: BZ, ASS, KR, EC; Collecting data: EC, MP; Completing data and statistical analysis: ASS; ECMO hub model: LP, MP; MD; Simulation scenarios: MD, PZ, IKP, AD, TK, LS, MS, WM, KB, ML, PL. All authors read and approved the final manuscript.

Abstract

BACKGROUND: Successful implementation of the medical technologies applied in life-threatening conditions, including extracorporeal membrane oxygenation (ECMO) requires appropriate preparation and training of medical personnel. The pandemic has accelerated the creation of new ECMO centers and has highlighted continuous training in adapting to new pandemic standards. To reach high standards of patients' care, we created the first of its kind, National Education Centre for Artificial Life Support (NEC-ALS) in 40 million inhabitants' country in the Central and Eastern Europe (CEE). The role of the Center is to test and promote the novel or commonly used procedures as well as to develop staff skills on management of patients needing ECMO.

METHOD: In 2020, nine approved and endorsed by ELSO courses of "Artificial Life Support with ECMO" were organized. Physicians participated in the three-day high-fidelity simulation-based training that was adapted to abide by the social distancing norms of the COVID-19 pandemic. Knowledge as well as crucial cognitive, behavioral and technical aspects (on a 5-point Likert scale) of management on ECMO were assessed before and after course completion. Moreover, the results of training in mechanical chest compression were also evaluated.

RESULTS: There were 115 participants (60% men) who were young (31-40 years). Majority of them (63%) were anesthesiologists or intensivists with more than 5-year clinical experience, but 54% had no previous ECMO experience. There was significant improvement in all cognitive, behavioral, and technical self-assessments after the course. Among aspects of management with ECMO that all increased significantly following the course, the most pronounced was related to the technical one (from approximately 1.0 to more 4.0 points). Knowledge scores significantly increased post-course from 11.4 ± SD to 13 ± SD (out of 15 points). The quality of manual chest compression relatively poor before course improved significantly after training.

CONCLUSIONS: Our course confirmed that simulation as an educational approach is invaluable not only in training and testing of novel or commonly used procedures, skills upgrading, but also in practicing very rare cases. The implementation of the education program during COVID-19 pandemic may be helpful in founding specialized Advanced Life Support centers and teams including mobile ones. The dedicated R&D Innovation Ecosystem established in the "ECMO for Greater Poland" program, with developed National Education Center can play a crucial role in the knowledge and know-how transfer but future research is needed.

Key words: ECMO; simulation, education, critical care, artificial life support, knowledge transfer, innovation cooperation, R&D alliances, business-academia cooperation.

1. Introduction

The history of the use of medical simulation as a training tool dates back to the 1950s, especially in the field of emergency techniques in critical conditions. In the last 50 years of the 20th century, an intensive development of training of resuscitation techniques based on mannequin simulation were observed. Simulation for extracorporeal therapy as a tool enabling structured and comprehensive training in ECMO (extracorporeal membrane oxygenation) support was first described by Anderson in 2006 [1]. Over the past 15 years, critical state medicine simulation techniques have been refined, from low-fidelity or computer models to high-fidelity models, to mimic realistic human responses. Simulation in extracorporeal techniques has also become a powerful tool in times of crisis. In 2009, in the face of the new AH1N1 pandemic, WHO recommended training based on medical simulation techniques as such that can bring the best measurable clinical outcomes [2-4].

High-fidelity simulation is a teaching methodology, a learning process that uses educational equipment from simple trainers for learning single tasks, through advanced mannequins, the so-called patient stimulators faithfully imitating the human and its parameters. The main task of medical simulation is to educate and improve patient safety. Advanced human simulators can realistically cough, vomit, and bleed. Fake blood causing medical staff real stress and the need for immediate action. The participants of high-fidelity medical simulation (using different techniques) can obtain better results and skills (mainly by learning-by doing) than in traditional education (Table 1) [5].

Table 1. Advantages of medical simulation and effective learning.

ADVANTAGES OF MEDICAL SIMULATION	FUNCTIONS OF HIGH-FIDELITY SIMULATION THAT LEAD TO EFFECTIVE LEARNING
• Use of real medical equipment under simulated conditions<br>• Practical exercises of invasive procedures<br>• Increasing the control of the accuracy of performed activities<br>• Constant repetition of practical skills, their assessment and analysis<br>• Enabling mistakes and showing their consequences in simulated conditions<br>• Avoiding risk to patients and learners<br>• Reduction of undesirable disturbances during exercise that may appear in the hospital or clinic<br>• The same scenario can be carried out for many students, thanks to which we achieve standardization of education<br>• Planning of clinical education based on student needs and curriculum, not patient availability<br>• Exposure to rare and complex clinical situations	• Integrating simulators into the general curriculum<br>• Clearly defined patterns and outcomes for learners using simulators<br>• Ability to repeat the skill exercises on the simulator multiple times<br>• Possibility of constructing exercises with increasing level of difficulty<br>• Ongoing feedback through the simulator during the learning process<br>• Adapting the simulator to complement a multidisciplinary teaching strategy<br>• The simulator should show the clinical variability<br>• Science should occur in a controlled environment<br>• Providing an individualized (apart from the team) educational process on the Simulator

• Drawing conclusions and summing up immediately after the end of the session during the debriefing<br>• The possibility of creating training scenarios that are very similar to real situations, thanks to which medical staff (students) can easily transfer the experience gained from training from theoretical conditions to the real situation in the future<br>• Validate the norms, standards and procedures by which student performance is assessed<br>• Possibility to diagnose educational needs on the basis of the obtained grade	

Use of ECMO as advanced life support is not a routine procedure in Poland in intensive care units, even of the highest reference. Apart from places in Poland where such treatment is carried out as part of bottom-up initiatives, there is no systemic solution. Additionally, there is lack of proper education programs that ensure easy access to this therapy [6-8]. Because the ECMO therapy organizational model is complex and expensive, we developed nationwide simulation based Artificial Life Support training in 2019 with cardiopulmonary resuscitation as an integral part of training program.

The planned educational system coincided with the vision of the new COVID-19 pandemic. In 2020, the specter of a pandemic was confirmed, which further emphasized the legitimacy and need for systematic training in the area of extracorporeal techniques. On the other hand, the deepening epidemiological situation forced significant restrictions in practical training while maintaining the sanitary regime.

1.1. Aim

The purpose of this study was to evaluate the results of training with the following scopes such as:

• development of medical skills and competence in the field of cardiovascular diseases, respiratory diseases resistant to conventional therapy with the additional possibility of training surgical skills necessary for ECLS,

• practical ECLS / ECMO application training, including the ability to master typical and rare emergency situations, by high fidelity simulation on clinically used equipment,

• improving the theoretical and practical skills of ECLS / ECMO in atypical, rare and difficult medical cases,

• improving trainings - supporting ECLS / ECMO competences for people with experience in this field as extended part of cardiopulmonary resuscitation (CPR),

• improving practical skills in high quality CPR, especially in chest compressions,

- assessment, validation, certification and re-certification of ECLS / ECMO skills.

2. Materials and Methods

In 2019, we founded the “Center of Artificial Life Support and Patient Safety” within a Medical Simulation Centre of the Poznan University of Medical Sciences and then developed in the first months a project devoted to “Artificial Life Support with ECMO” as National Education Centre for Artificial Life Support (NEC-ALS). The center is equipped with modern simulation education tools and classroom technology. The training program includes theoretical and practical components related to advanced ECLS (Extracorporeal Life Support)/ECMO therapy techniques, as well as workshops and immersive scenario-based simulations. The training program rely on valid national respiratory failure and Extracorporeal Life Support Organization (ELSO) guidelines. Of note, this is endorsed by ELSO, as first one in Poland.

The program is offered for 264 physicians specializing in anesthesia and intensive care, cardiac surgery, cardiology, thoracic surgery, vascular surgery, transplant medicine, emergency medicine, and other physicians in training from all over Poland to deploy and manage ECMO. The scope of the training is a theoretical and practical introduction to advanced extracorporeal life support (ECLS)/ECMO therapy techniques based on cardiopulmonary resuscitation guidelines, practical workshops and simulations of high fidelity. Another primary goal is to consolidate physicians’ awareness regarding the availability of ECLS technology that may be used to save patients after exhaustion of conventional therapy. The program was launched in 2019 with 3 courses and ELSO endorsement. However, during the difficult period of the COVID-19 pandemic in 2020, 9 editions were carried out for 115 (course IV to XII) participants and that part of project has been analyzed. The subject matter of the course clearly corresponded to the epidemiological and training needs of the country. Most of the participants represented centers assuming the primary burden on critically ill patients as a consequence of the COVID-19 epidemic, including extracorporeal life support techniques, high-quality cardiopulmonary resuscitation, and transportation of critically ill patients.

2.1. Participants

The group of 115 physicians participated in 9 editions of course in 2020 – specialists and residents in anesthesiology and intensive care, cardiology, cardiac surgery, thoracic surgery, vascular surgery, transplant medicine, and emergency medicine. The recruitment was voluntary and open to all physicians in Poland, regardless of their experience.

2.2. Form of education

The postgraduate education was formulated in - 3-day course, where 50% of educational hours were implemented in the form of workshops and classes in simulated conditions - 25 didactic hours spread over three days during a one weekend meeting. One week before the courses every participant was obligated to perform pre-course knowledge tests and self-assessment forms, then e-learning platform including 5 lessons of 20 slides was open for every person.

2.3. Pre-test and pre-forms

Proposed by ELSO pre-course forms consist of demographic information's, type of physician's specialization, working place and ECMO experience and additionally 11 cognitive, 4 behavioral and 6 technical self-assessment questions (on a 5 point Likert scale), focused on aspects of managing patients supported on ECMO. Knowledge test consists of 15 multiple choice questions on the theoretical base of artificial life support with extracorporeal techniques.

2.4. Scope of the program

Scope of knowledge, objectives and skills are included in Supplementary material 1. The frame program (Supplementary material 2) of 3 days including first day with 6 lectures, second with water drills, priming; ultrasound visualization and 7 high-fidelity scenarios, third day including transportation workshops; 3 high-fidelity scenarios and theoretical and practice examination (Supplementary material 3). Two different ECMO platforms; Cardiohelp (Getinge, Rastatt, Germany) and Novalung (Fresenius, Bad Homburg, Germany) were used.

2.5. Post-test and post-forms

Proposed by ELSO post-course forms consist of 11 cognitive, 4 behavioral and 6 technical self-assessment questions similar to the pre-course questions. Post-course knowledge test consisted of 15 multiple choice questions on the theoretical basis of artificial life support with extracorporeal techniques. There were no repeat questions in the pre and post-course knowledge tests. Additionally, course assessment and educational benefits were provided in 6 areas.

2.6. Practice part

Consist of individual chest compression tests attempted at individual resuscitation stations Resusci Anne QCPR (Laerdal, Norway) in the course of the test A: 2-minute trial without preview – verification of the current state and B: 2-minute test with QCPR preview (quality improvement). Second part consists of 15 minutes sessions of mechanical chest compression devices use – LUCAS (Stryker, USA).

During chest compression following parameters were monitored: compression rate, depth and recoil percentage.

The last part included exam simulation scenario (pump failure in veno-arterial VA ECMO, gas supply failure on veno-venous VV ECMO), where every participant had 8 min to emergency intervention in simulated ICU room. The proposed by ELSO forms evaluated cognitive, behavioral and technical skills in addition to times to critical action completion in each scenario.

2.7. Teaching materials

In pre-course time the 100 slides introduction presentation in e-learning form – www.ecmo.pl was opened. Additional materials included ELSO base (www.elso.org) and recommended books: "Red Book 5th Edition" (ELSO) and "ECMO Specialist Training Manual" (ELSO) – both free for students.

2.8. Statistical analysis

Descriptive statistics of measurable variables were performed during the analysis. Due to the non-normal distribution among the variables, nonparametric tests were used and data were presented as median [interquadrile range]. For normal distribution data were presented as mean±SD. To check the significance of differences Mann-Whitney U test and Wilcoxon pairwise rank test were used as appropriate. A value of $p<0.05$ was considered as statistically significant. Statistical analysis was performed in STATISTICA Application, version 13 using the ELSO database.

3. Results

3.1. Participants

In 2020, 115 physicians took part in the courses and all of them completed both pre and post-course documents. The predominant motivation to be trained was a will for self-improvement (n=103), in 10 others recommendation and only in 2 superior's order. The participant demographics, specialty and hospital settings with clinical practice and ECMO experience are included in Table 2.

Table 2. Participant demographics, specialty and hospital settings with clinical practice and ECMO experience.

Variable		Count	Percent
Gender	Female	47	40.9
	Male	68	59.1
Age group	21-30 years	15	13.0
	31-40 years	60	52.2
	41-50 years	26	22.6
	51-60 years	13	11.3
	>60 years	1	0.9
Specialty	Other	3	2.61
	Physician – Anesthesiologist	56	48.7
	Physician – Cardiac Surgeon	6	5.2

	Physician – Cardiologist	9	7.8
	Physician – in trainee	23	19.0
	Physician – Intensivist	17	14.8
	Physician – Trauma Surgeon	1	0.9
	Physician – Vascular Surgeon	1	0.9
Hospital setting	Academic Hospital	55	47.8
	Community Hospital	40	34.8
	Government Hospital	11	9.6
	Other	9	7.8
Patient population	Adult	94	81.7
	Neonatal, Adult	1	0.9
	Pediatric	5	4.3
	Pediatric, Adult	4	3.5
	Pediatric, Neonatal	2	1.7
	Pediatric, Neonatal, Adult	9	7.8
Clinical practice duration	<5 years	20	17.4
	>20 years	20	17.4
	10-15 years	16	13.9
	15-20 years	19	16.5
	5-10 years	40	34.8
ECMO experience	<6 months	18	15.6
	>5 years	4	3.5
	0 months	62	53.9
	1-2 years	14	12.2
	2-5 years	7	6.1
	6-12 months	10	8.7
Hospital ECMO experience	<6 months	4	3.5
	6-12 months	3	2.6
	1-2 years	12	10.4
	2-5 years	21	18.3
	> 5 years	34	29.6
	We do not have ECMO and are not planning to	8	7.0
	We do not have ECMO but are planning to	33	28.7
Annual ECMO volume	<10 patients a year	86	74.8
	10-20 patients a year	23	20.0
	20-50 patients a year	4	3.5
	>50 patients a year	2	1.7
ECMO modes	Both VV & VA	56	48.7
	VA	4	3.5
	VV	21	18.3
	None	34	29.6
ECMO platform used	Maquet Cardiohlep	33	28.7
	Maquet Rotaflow	32	27.8
	Sorin Revolution	4	3.5
	Xenios Fresenius	11	9.6
	None	35	30.4
ECMO capability	Can cannulate and care for ECMO patients	75	65.2

	Can cannulate patients onto ECMO but then refer to outside institutions	11	9.6
	No ability to cannulate for ECMO but can refer to outside institutions	20	17.4
	No ability to cannulate or refer for ECMO	8	7.0

3.2. Cardiopulmonary assessments

In total assessments in men activity chest compression depth was (56mm) in relation to women (46mm). Both groups pressed with too fast rate 120/min with not proper chest return to its original value (expansion) – chest recoil – 61.5% - Table 3.

Table 3. Manual chest compression assessments with and without feedback device.

**Chest compressions in CPR**	**Group**	**female**	**male**
mean depth (mm)	51.2	46.4	56.0
mean rate (/min)	123.3	124.5	122.2
chest recoil (%)	61.5	60.6	62.3
**Chest compressions in CPR with feedback device**	**Group**	**female**	**male**
mean depth (mm)	56.8	53.3	58.3
mean rate (/min)	107.8	104.4	111.3
chest recoil (%)	99.1	99.8	98.4

3.3. ECMO cognitive assessments

In all detailed aspects of cognitive assessment, improvement was observed after the course. Therefore, the mean value for all aspects taken together was significantly lower before (less than 4.0) than after the courses (more than 4.5) ($p<0.001$). Of note, medians of the baseline values were either 4 (in eight out of 11 aspects) or 3. After the course, all but two reached maximal value of 5 – Figure 1.

3.4. ECMO behavioral assessments

A courses imroved significantly self-confidence of participants to play different roles in ECMO teams but also to react in emergency situations. Before training, the median value for leading ECMO team role was the

lowest (1 point) whereas after the course increased by 3 points to reach 4. Mean value of all aspects of behavioral assessment increased 2-fold, from 2 to 4 ($p < 0.001$) – Figure 2.

3.5. ECMO technical assessments

The most significant improvement in technical skills was observed after the course. Before the latter one, only single physicians have any experience with ECMO devices. Therefore, the median values in all 6 aspects of technical experience were minimal. Following two days devoted predominantly for developing practical abilities, post-course technical assessment revealed more than 4-fold increase in expertise level in this area. Among all aspects of the aforementioned evaluation, the most pronounced increase was noted for management of pump failure from 1 to 5 – Figure 3.

3.6. ECMO knowledge assessments

It corresponded to the cognitive assessment. Pre-course knowledge regarding ECMO theory assesment tests presented mean score 11.4 out of 15 (76.0%). The results of post-course test were significantly higher than before training and mean score increased to approximately 13 points (85.3%) – Table 4 and Figure 4.

Table 4. Pre and post-course knowledge assessments (maximum score 15).

Test	Mean	SD	Min	Max
**pre-course**	11.4	2.81	2	15
**post-course**	12.8	1.6	8	15

3.7. ECMO simulation assessments

In 115 group of participants, 57 completed VA pump failure sim, and rest VV gas failure scenario. The main noticed cognitive reaction was the lack of proper patient support in both scenarios – emergency ventilator settings and vasopressors.

In VA pump failure scenario the mean time to change power supply to hand crank was 200 seconds (80:450) – Figure 5A. In the VV gas failure scenario mean time to recognize gas supply problem was 55 seconds (40:115) – Figure 5B.

4. Discussion

In 2020 our center was one of the few in the world, despite COVID-19 pandemic, doing courses in the online (lectures) and stationary model (hybrid). In order to adapt to COVID-19 pandemic restrictions we offered simultaneous knowledge sharing and division into smaller teams (internal innovations in COVID-19 pandemic). The relationship number of students for participants has been changed – during COVID-19 pandemic there were maximum 4 people per room and 1 instructor in theoretical part and debriefing and 2 instructors for every 2 participants in ECMO technical skills and 1 for 4 during simulations. It was possible thanks to simultaneous simulations and debriefings in three 4-person subgroups with innovative audio-visual solutions.

4.1. Chest compressions during CPR

Chest compressions (CC) are an essential part of efficient high-quality cardiopulmonary resuscitation and ensure proper flow in the coronary and cerebral vessels. The CC quality criteria include proper rate and adequate depth of CCs, full chest recoil, and minimal interruptions during CCs and are included in European Resuscitation Council guidelines [9-12].

Despite the fact that the vast majority of participants were experienced clinicians, chest compressions require a definite improvement. Professionals performed too fast and too superficial compressions with a low percentage of recoil. The last parameter is sometimes underestimated but it improves blood flow and maintains the compression to decompression relationship 1:1. Using chest compressions feedback devices improved chest compression quality in every parameter. The self-control is the most important trigger in that improvement and adaptation to the applicable guidelines.

The use of MACC allows for the systematization of CC by parameters required in the guidelines, safety patient transportation during CPR and is convenient in small rescue teams. All physicians justified to have an MACC device both in out-of-hospital conditions (emergency medical teams) and in-hospital CPR. The training influenced the achievement of the learning effect, which is the mastery of the MACC connection skills during a short 15-minute practice session, with particular attention to the correct position of the compression piston in relation to the chest [9-12].

4.2. Extracorporeal support trainee

We are not surprised that the issue of weaning from either VV or VA configuration was the most difficult aspect of ECMO therapy revealed by our questionnaires in cognitive and behavioral

assessments. The issue of cessation (in both a positive and negative sense) of support of any failing organ is the most crucial and challenging point in the intensive care and therapy [13, 14]. Although, there are recommendations regarding the aforementioned situations published by different working groups and societies, there is still a lot of place for independent decision of physician responsible for patient treatment [15, 16]. These decisions are always not straightforward and many factors may have impact on the final clinical outcome. Of importance is experience in a given technology. During three-day course it is possible to extend knowledge but, in our opinion, only numerous and systematic real-life clinical applications enable to reach a true level of expertise and self-confidence (5 points).

Among behavioral aspects of management with ECMO the best improvement was revealed in playing a role of leader of the team. We considered it as great achievement of our course. One knows it is much easier to be a member than to be in charge of a leader of any novel and cutting-edge technologies. If we are immersed in ECMO therapy we are probably more willing to be a leader of team. In our opinion this improvement results from gained during three-day training not only knowledge but also practical as well as manual skills.

The most significant improvement after the course was observed in technical skills. Before the latter one, only single physicians have any experience with ECMO devices. Of note, during the course special stress was put on development of technical skills that was also reflected by technical assessment before and after training. It is also supporting our enthusiasm for high fidelity simulators as tools featured by safety and enormous educational value [7, 8]. We must express our satisfaction that management of pump failure achieved the best score after the course. It is one of the most serious critical situations during ECMO application. Therefore, we did not only repeat many times during workshops how to solve such problem but also pump failure was chosen for one of the practical tests at the end of our course. The second one was the gas supply failure scenario, which was also based on our real-life experience.

Knowledge assessments proved good theoretical background of participants. Pre-course knowledge regarding ECMO theory was more than satisfying. It may result from a fact that the vast majority of physicians pointed self-improvement as the predominant motivation to participate in training. Therefore, probably most of them were especially interested in ECMO and had read research articles or other on-line publications regarding this device before already before our course. Moreover, we cannot exclude that current pandemic and real problems with critically ill requiring invasive support made many intensivists know more about artificial life support methods. Even in spite of good pre-course knowledge, out training was also proved to be useful in a such aspect for the vast majority of participating physicians.

4.3. Translational simulation in advanced life support

In many fields, especially in cardiopulmonary resuscitation simulation techniques offers promising outcomes and have been proved in many publications. High fidelity ECMO training unfortunately have rarely been studied and published reports did not answer to the question if the use of simulators improved outcomes and/or decreased the number of serious adverse events.

Zakhary et al. presented the benefits of a practical simulation-based ECMO training in improving ECMO skills and significant reduction in delay to undertake emergency actions. This study is the first randomized controlled trial evaluating the application of simulation in ECMO education [17]. Puslecki et al. in their papers described the employment of medical simulations as a form of creating and testing tool of difficult and rare procedures. The effectiveness of translational medicine is confirmed by authors in many areas of application of extracorporeal techniques and in transporting critically ill patients. The simulation activities presented by the authors allowed for the creation of algorithms for proceedings in the regional program "ECMO for Greater Poland" [5-12,18,19]. Additional studies are needed to determine if simulation training will reduce complication rate and improve patients' outcomes [20-22]. The ELSO, organization established in 1989 supports health care professionals and scientists who are involved in extracorporeal techniques including guidelines creation and providing dedicated endorsed courses in artificial support subjects [23].

Patient management in critical state is definitely a team work and ECMO requires a multidisciplinary healthcare team. Sin et al. proved that simulation training that focuses on crisis resource management and proper communication is well suited to these training needs. Moreover, institutional commitment to providing public support as well as human resources is essential to the success of ECMO training programs [24]. The nurses training is crucial part in intensive care especially in sudden cardiac arrest, dynamic patient state deterioration or management with extracorporeal support. In isolated epidemic area, "hot zones" nurses are the first recipient of urgent situations and simulation training allows them to be properly prepared for such interventions [25].

In USA 46% of certified ELSO centers had an ECMO simulation program, whereas 26% report a program is in development. Sixty-three percent use simulation for summative assessment, and 76% have multidisciplinary training. Access to a simulation center allows for structured training and largest centers provide mannequin-based ECMO simulation (the most common scenarios include pump failure (93%), oxygenator failure (90%), and circuit rupture (76%)) [26].

The most important for patient with ECMO support transportation safety is well trained and cooperating dedicated ECMO team. In Broman et al's survey, 14 of 15 centers performed programs for structured training – 33% provide full ECMO clinical and ECMO transport training, (i.e., simulations,

rescue training aircraft); 60% regular ECMO simulations, 20% annual transport simulations, 33% others perform ECMO pump training and in-house simulations [19].

4.4. ECMO in Poland before 2020

Extracorporeal techniques in VV configuration in the Polish intensive care units were not routine procedures, except few centers, that tried to realize Hub and Spoke concept: Cracow, Lublin, Warszawa, Poznan, Opole. There were no systemic solutions that would ensure easy access to this therapy because there was no dedicated ECMO transportation. Additionally, there was a lack of data regarding use (exact numbers and places) of VV-ECMO systems. More information was available on VA-ECMO support in pediatric and adult cardiac surgery centers (more than 45 centers).

”ECMO for Greater Poland” is an example of innovative cooperation HUB concept developed in 3.5 million inhabitant’s region. Created in 2015 as nation’s first, implemented all program arms to improve support in patients with severe reversible respiratory failure (RRF), hypothermia, critical states resulting in heart failure due to cardiac arrest, cardiogenic shock, or acute intoxication and promotion of donor after circulatory death (DCD) strategy in selected organ donor cases, after unsuccessful lifesaving treatment, to achieve organ recovery. In the last 4 years, many transportations of ECMO-supported critically ill patients were realized. Before launching the program, the ECMO support application in this region was incidental, despite devices availability [6-8,18,19].

4.5. New pandemic reality

COVID-19 pandemic outbreak, a global critical situation in the whole healthcare sector, has forced to reorientation of existing procedures with the use of personal protecting equipment (PPE) , new and modified skills of medical staff and development of medical infrastructure and facilities. Additionally, such dynamic situation has worsened quality of reporting and analyzing current data regarding COVID-19 infections. Taking into account the new challenges which affect the healthcare system (new virus mutations, desire for new and modified vaccines) different stakeholders involved in healthcare sector need to cooperate and share knowledge, which will help COVID-19 pandemic faster defeating [27, 28].

In order to improve the patient care we should take into consideration new technological solutions (digital Health, medHealth, telehealth and telemedicine, and personalized medicine) which can be used to protect healthcare workers, quickly detect and prevent the spread of COVID-19, and improve intensive care (better diagnose and monitor of the patients) [28]. Boosting innovation

cooperation and R&D alliances between academia, institutions and business can significantly increase the likelihood of creating better medical therapy for patients. The results of such cooperation can be safe vaccines for COVID-19 which are very important in pandemic defeating [28].

Because of the lack of the training in clinical areas of medical personnel due to COVID-19 pandemic we can observe the greater use of medical simulation, using high-fidelity one with different techniques like: trainers, HPS – Human Patient Simulator, BLS – Basic Life Support and ALS – Advanced Life Support computer simulation, SP – Standardized Patients and debriefing. We can find a lot of medical centers in the world affected by pandemic situation offering training with the use of medical simulation for the staff, which are the most exposed to COVID-19 pandemic. For sure COVID-19 pandemic forced changes in the area of critical care, that is why we need to be more open to cooperation using local potential and partners in the development of better therapies for patients [19, 27, 29, 31-39].

The new sudden COVID-19 pandemic and its dynamics in 2020 accelerated the implementation of extracorporeal techniques in many countries where it had not been widely used so far. A specific group of critically ill, mechanically ventilated patients requires extended support with possible survival at VV ECMO 50-60% [23]. The rapid growth of teams dedicated to the treatment of patients with critical respiratory failure and the growing number of extracorporeal devices provoke the necessary training in the areas of application of extracorporeal techniques including patient transportation.

In addition, the need to efficiently manage PPE and human resources in the times of crisis, with the coexisting threat of coronavirus and without causal effective treatment, requires the reorganization of all sectors of social life, including reprogramming the procedures used so far in prevention and medical therapy. Dynamic increase in numbers of infected and/or suspected patients, as well as the need to inhibit virus transmission, require a change of approach in even the fundamental techniques of dealing with critical states, such as cardiopulmonary resuscitation or traditional non epidemic approach for every patient [30-36].

4.6. ECMO in Poland in 2020 pandemic year

Despite the lack of dedicated solutions in VV support centers and in developing pandemic some casual solutions were prepared, and 5 dedicated centers for VV ECMO support in COVID patients (Lublin, Warsaw, Cracow, Gdansk, Wroclaw). Moreover, the air transportation concept was created (HEMS – Helicopter Emergency Medical Service – helicopters and planes) in Poland, and for several months transport by helicopter has been available 24 hours a day in five Polish HEMS stations. There is still lack of any kind confirmed number of treated cases or patient’s outcomes. During last month’s

few ground (Lublin, Cracow, Zabrze, Warszawa, Poznan, Opole, Wroclaw) and air transfer of ECMO-supported COVID-19 patients were performed [19]. In the last months "ECMO for Greater Poland" mobile ECMO team with dedicated ambulance recently performed four transfers including one with COVID-19 suspected patient, one post COVID-19 pulmonary failure case and 2 actively infected subjects [19].

4.7. Center for Artificial Life Support and Patient Safety

"ECMO for Greater Poland" as Artificial Life Support HUB coordinator body in the dedicated R&D Innovation Ecosystem (Figure 6) also has an educational arm of the project [19, 29]. The additional knowledge dissemination in the „ECMO for Greater Poland" Program is realized mainly through different scientific articles (also in interdisciplinary teams) in reputed scientific journals, presentations on different national and international conferences, as well as through organizing the international conferences „ECMO for Greater Poland" in Poznań (Poland) in 2017 and 2019, which met with high recognition in the community.

Education center makes it possible to improve the existing procedures, monitor individual ECMO cases, and constantly improve the qualifications of all ecosystem participants. It is worth mention, that as a part of the education center, there is ongoing R&D cooperation focused on developing an ECMO human patient simulator and IT software (with dedicated mobile application, both for Android and IOS systems). This center is unique in Poland and in the Central and Eastern Europe region – under ELSO auspicious. The authority of this organization is still improved in recent difficult year and resulted in the proper guidelines in intensive and critical care of ECMO supported patients [19]. Thanks to ELSO's trust, the project gained recognition from the Organization and during the first meeting in 2019 it was positively certified and obtained the status of ELSO-endorsed course.

Initiative was continued despite developing COVID-19 pandemic with acute epidemiological regime and participant safety in 2020 in 9 sessions for 115 physicians from whole country. In project authors' opinion it is a valuable contribution to the development of highly qualified personnel and fills the gap in the field of extracorporeal techniques in Poland.

4.8. Development of Innovation Cooperation and Knowledge Transfer in the „ECMO for Greater Poland" Program in 2020

In order to improve the quality of our Educational Program we constantly monitor, evaluate and raise our competences according to current challenging times. As only one ELSO certified center in Poland and in the Central and Eastern Europe we can observe huge demand for our course not only in

Poland, but also in other countries (inquiries from the Central and Eastern Europe (CEE) countries like Lithuania and Latvia), especially because of COVID-19 pandemic. Despite the lockdown including of the contact trainings, the course was adapted to the epidemiological needs and in accordance with the current restrictions the 115 participants completed courses.

Nowadays we can also observe the very important result of ECMO program – popularization of extracorporeal techniques not only in Greater Poland but also in the whole country and abroad. Such a trend is noticeable especially in developed countries, which have equipped their centers with the necessary devices for extracorporeal support over time. However, there were still obstacles in the form of a lack of separate financing, dedicated training, especially in practical skills. It was one of the reasons that's why we have decided to launch such a program and educational course. It is worth to mention the international nature of the course – because of the cooperation with worldwide organization – ELSO.

Thanks to knowledge and know-how transfer in the „ECMO for Greater Poland" program and knowledge diffusion it was possible to create new ECMO Teams in different parts of Poland, which are very desirable due to the dynamic development of the COVID-19 pandemic in our country recently. Without „ECMO for Greater Poland" Program, involvement of different stakeholders in R&D Innovation Ecosystem, as well as innovation cooperation in the whole program, it would not be possible to absorb the knowledge from the program in different part of Poland in such a short time, especially in pandemic difficult time in 2020. The devoted Education Center and Platform are a great solution in this matter – offer both teaching, learning and assessing services (according to Program Learning Outcomes) for participants of Educational Program, with ELSO support.

4.9. Limitations

Up to now we can present only early results of our course and we must wait for at least some months or years to know how training in ALS impacted on clinical practice and ECMO applications. In spite of this limitation, the short-term results of developed concept in "Center of Artificial Life Support and Patient Safety" with ELSO cooperation are very promising. In the future, the authors together with ELSO plan to carry out a prospective assessment of the long-term outcomes in the terms of number of new and active ECMO centres in Poland, the total number of supported patients and successful transportations as well as the final outcomes of the treated individuals. Moreover, having practice in ECMO applications, we are aware how important is good cooperation with many other members of medical personnel and emergency system. This course was only for physicians. Of note, during training we appeal for participants to create their own teams and to play by them role of leaders. The significant improvement in behavioral assessment, particular leader of ECMO team, gives hope for successful involvement other medical professionals.

5. Conclusions

Our course confirmed the simulation as an educational approach is valuable not only in training and testing of novel or commonly used procedures, skills upgrading, but also in practicing very rare cases. The implementation of the education program during COVID-19 pandemic may be helpful in founding specialized ECMO centers and teams including mobile ones.

The dedicated R&D Innovation Ecosystem established in the "ECMO for Greater Poland" program, with developed National Education Center can play a crucial role in the knowledge and know-how transfer. More research is needed to assess the sustainability of training and its impact on the quality of health care.

Abbreviations: ALS – artificial life support; CC – chest compression; CPR – cardiopulmonary resuscitation; eCPR – extended cardiopulmonary resuscitation; COVID-19 – coronavirus disease; ECLS – extracorporeal life support; ECMO – extracorporeal membrane oxygenation; ELSO – Extracorporeal Life Support Organization; HEMS – Helicopter Emergency Medical Service; HPS – human patient simulator; ICU – intensive care unit; MACC – mechanical automated chest compression device; PPE – personal protective equipment; RRF – reversible respiratory failure; R&D – research and development; VV – venovenous; VA – venoarterial; DCD – donor after circulatory death; WHO – World Health Organization

Funding: The project was awarded funding from a POWER competitive national grant (POWR.05.04.00-IP.05-00-006/18) by the Polish Ministry of Health for a total of 2,750,000 USD (PLN 10,974,708.60). Main reason was to develop a course about "Artificial Life Support with ECMO" offered to 264 physicians from Poland implemented in 2019-2021 at the Poznan University of Medical Sciences (PUMS). This paper includes also findings from the research project financed by the research grant of the National Science Centre (Poland) awarded based on the decision no. DEC-2015/19/D/HS4/0041.

Ahmed S. Said acknowledges research support from the Children's Discovery Institute Faculty Development Award at Washington University in St. Louis.

Acknowledgments: Special thanks from the authors to "ECMO for Greater Poland" Program co-authors and co-creators: Marcin Ligowski, Marek Dąbrowski, Sebastian Stefaniak, Agata Dąbrowska, Małgorzata Ładzińska, Piotr Ładziński, Konrad Baumgart, Lidia Szlanga, Tomasz Kłosiewicz, Maciej Sip, Radosław Zalewski, Piotr Ziemak, Norbert Toboła, Ilona Kiel-Puślecka, Magdalena Wieczorek, Michał Kiel, Marcin Zieliński, Aleksander Pawlak, Roland Podlewski, Lukasz Gąsiorowski, Marek Karczewski, Krzysztof Kusza, Wojciech Mrówczyński, Luksza Szarpak; Bartłomiej Perek, Marek Jemielity.

Special thanks to ELSO organization: Bishoy Zakhary, Ahmed S. Said, Kollengode Ramanathan, Elaine Cooley, Tammy Friedrich, Justyna Swol, Mark Ogino.

Ethics approval: According to the rules of Local Bioethical Committee of Poznan University of Medical Sciences the Statement of Ethics Approval is not required.

Institutional Review Board Statement: Not applicable.

Informed Consent Statement: Not applicable.

Data Availability: The data used to support the findings of this study are available from the corresponding author upon request.

Disclosure: The authors alone are responsible for the content and writing of this paper.

Conflicts of Interest: The authors declare that they have no conflicts of interest.

Bibliography

1. Anderson, J.M.; Boyle, K.B.; Murphy, A.A.; Yaeger, K.A.; Le Flore, J.; Halamek, L.P. Simulating extracorporeal membrane oxygenation emergencies to improve human performance. Part I: methodologic and technologic innovations. *Simul Healthc* **2006**, *1*, 220–227. https://doi.org/10.1097/01.SIH.0000243550.24391.ce.
2. Brazzi, L.; Lissoni, A.; Panigada, M.; Bottino, N.; Patroniti, N.; Pappalardo, F.; Gattinoni, L. Simulation-based training of extracorporeal membrane oxygenation during H1N1 influenza pandemic: the Italian experience. *Simul Healthc* **2012**, 7, 32–34. https://doi. org/10.1097/SIH.0b013e31823ebccb.
3. Sakamoto S. Simulation-based training for handling extracorporeal membrane oxygenation emergencies. *J Thorac Dis* **2017**, *9*, 3649–3451. https://doi.org/10.21037/ jtd.2017.09.102.
4. Banfi, C.; Bendjelid, K.; Giraud, R. High-fidelity simulation for extracorporeal membrane oxygenation training, utile or futile? *J Thorac Dis* **2017**, *9*, 4283–4295. https://doi. org/10.21037/jtd.2017.10.54.M.
5. www.ecmo.pl
6. Puslecki, M.; Ligowski, M.; Dabrowski, M.; Telec, W.; Perek, B.; Jemielity, M. "ECMO for Greater Poland": a unique regional program for extracorporeal life support. *Pol Arch Intern Med* **2017**, *127*, 567–568. Doi: 10.20452/pamw.4082.
7. Puślecki, M., Ligowski, M., Dąbrowski, M., Stefaniak, S.; Ladzińska, M.; Ladziński, P.; Pawlak, A.; Zieliński, M.; Dąbrowska, A.; Artyńska, A. et. l.. BEST Lif– " "Bringing ECMO Simulation To Li"e– - how medical simulation improved a regional ECMO program. *Artif Organs* **2018**, *42*, 1052-1061. doi: 10.1111/aor.13332.
8. Puślecki, M.; Ligowski, M.; Stefaniak, S.; Zieliński, M.; Pawlak, A.; Dąbrowski, M.; Kłosiewicz, T.; Sip, M.; Karczewski, M.; Małkiewicz, T. el.al. Using simulation to create a unique regional ECMO program for the Greater Poland region. *Qatar Med J* **2017**, 79. doi: 10.1016/j.ajem.2018.04.030.
9. Kłosiewicz, T.; Puślecki, M.; Zalewski, R.; Sip, M.; Perek, B. Impact of automatic chest compression devices in out-of-hospital cardiac arrest. *J Thorac Dis* **2020**, *12*, 2220-2227. doi: 10.21037/jtd.2020.04.25.
10. Zalewski, R.; Puślecki, M.; Szarpak, Ł.; Kłosiewicz, T.; Dąbrowski, M.; Perek, B. Impact of Application of Multifunction Electrode (MFE) Pads on Cardiopulmonary Resuscitation Quality. *Emerg Med Int* **2020**, *19*, 2020:2675214. doi: 10.1155/2020/2675214.
11. Sip, M.; Puslecki, M.; Dabrowski, M.; Klosiewicz, T.; Zalewski, R.; Ligowski, M.; Goszczynska, E.; Paprocki, C.; Grygier, M.; Lesiak,l..; et al. Extended cardiopulmonary resuscitation: from high fidelity simulation scenario to the first clinical applications in Poznan out-of-hospital cardiac arrest program. *Perfusion* **2020** Dec 16:267659120981811. doi: 10.1177/0267659120981811.
12. Sip, M.; Puślecki, M.; Kłosiewicz, T.; Zalewski, R.; Dąbrowski, M.; Ligowski, M.; Goszczyńska, E.; Paprocki, C; Grygier, M.; Lesial. M.; et al. A concept for the development of a pioneer regional Out-of-Hospital Cardiac Arrest Program to improve patient outcomes. *Kardiol Pol* **2020**, *25*, 875-881. doi: 10.33963/KP.15433.
13. Rose, L. Strategies for weaning from mechanical ventilation: a state of the art review. *Intensive Crit Care Nurs* **2015,** *31*, 189-195. https://doi: 10.1016/j.iccn.2015.07.003.
14. Haldenwang, P.L.; Baumann, A.; Elghannam, M.; Schlömicher, M.; Buchwald, D.; Klak, K.; Strauch, J.T. Two-stage weaning strategy using veno-veno-arterial perfusion for patients on extracorporeal membrane oxygenation following cardiogenic shock. *Perfusion* **2019**, *34*, 689-695. https://doi: 10.1177/0267659119841862.
15. Sangalli, F.; Bellani, G.; Affronti A.; Volpi, F.; Feri, M.; Marini, M.; Quacquarelli, A.; Vitale, D.; Guarracino, F. Levosimendan to facilitate weaning from cardiorespiratory support in critically ill patients: current evidence and future directions. *Minerva Anestesiol* **2020,** *86*, 645-651. https://doi: 10.23736/S0375-9393.20.14219-6.
16. Perkins, G.D.; Mistry, D.; Gates, S.; Gao, F.; Snelson, C.; Hart, N.; Camporota, L.; Varley, J.; Carle, C.; Paramasivam, E.; et al Breathe Collaborators. Effect of Protocolized Weaning With Early Extubation to Noninvasive Ventilation vs Invasive Weaning on Time to Liberation From Mechanical Ventilation Among Patients With Respiratory Failure: The Breathe Randomized Clinical Trial. *JAMA*. **2018**, *320*, 1881-1888. https://doi: 10.1001/jama.2018.13763.
17. Zakhary, B.M.; Kam, L.M.; Kaufman, B.S.; Felner, K.J. The Utility of High-Fidelity Simulation for Training Critical Care Fellows in the Management of Extracorporeal Membrane Oxygenation Emergencies: A Randomized Controlled Trial. *Crit Care Med* **2017**, *45*, 1367-1373. doi: 10.1097/CCM.0000000000002437.
18. Puślecki, M.; Ligowski, M.; Dąbrowski, M.; Stefaniak, S.; Ładzińska, M.; Pawlak, A.; Zieliński, M.; Szarpak, Ł.; Perek, B.; Jemielity, M. Development of regional extracorporeal life support system: The importance of innovative simulation training. *Am J Emerg Med* 2019, *37*, 19-26. doi: 10.1016/j.ajem.2018.04.030.

19. Puslecki, M.; Baumgart, K.; Ligowski, M.; Dabrowski, M.; Stefaniak, S.; Ladzinska, M.; Goszczynska, E.; Marcinkowski, P.; Olasinska-Wisniewska, A.; Klosiewicz, T. et al. Patient Safety during ECMO Transportation: Single Center Experience and Literature Review. *Emerg Med International* **2021** https://doi.org/10.1155/2021/6633208.
20. Brum, R.; Rajani, R.; Gelandt, E.; Morgan, L.; Raguseelan, N.; Butt, S.;Nelmes, D.; Auzinger, G.; Broughton, S. Simulation training for extracorporeal membrane oxygenation. *Ann Card Anaesth* **2015**, *18*, 185–90. doi: 10.4103/0971-9784.154472.
21. Burton, K.S.; Pendergrass, T.L.; Byczkowski, T.L.; Taylor, R.G.; Moyer, M.R.; Falcone, R.A.; Geis, G.L. Impact of simulation-based extracorporeal membrane oxygenation training in the simulation laboratory and clinical environment. *Simul Healthc* **2011**, *6*, 284–291. doi: 10.1097/SIH.0b013e31821dfcea.
22. Fehr, J.J.; Shepard, M.; McBride, M.E.; Mehegan, M.; Reddy, K.; Murray, D.J; Boulet, J.R. Simulation-based assessment of ECMO clinical specialists. *Simul Healthc* **2016**, *11*, 194–199. doi: 10.1097/sih.0000000000000153.
23. Extracorporeal Life Support Organization; https://www.elso.org (2020). Accessed 5 March 2021.
24. Sin, S.W.C.; Ng, P.Y.; Ngai, W.C.W.; Lai, P.C.K.; Mok, A.Y.T.; Chan, R.W.K. Simulation training for crises during venoarterial extracorporeal membrane oxygenation. *J Thorac Dis* **2019**, *11*, 2144-2152. doi: 10.21037/jtd.2019.04.54.
25. Fouilloux, V.; Gran, C.; Guervilly, C.; Breaud, J.; El Louali, F.; Rostini, P. Impact of education and training course for ECMO patients based on high-fidelity simulation: a pilot study dedicated to ICU nurses. *Perfusion* **2019**, *34*, 29-34. doi: 10.1177/0267659118789824.
26. Weems, M.F.; Friedlich, P.S.; Nelson, L.P.; Rake, A.J.; Klee, L.; Stein, J.E.; Stavroudis, T.A. The Role of Extracorporeal Membrane Oxygenation Simulation Training at Extracorporeal Life Support Organization Centers in the United States. *Simul Healthc* **2017**, *12*, 233-239. doi: 10.1097/SIH.0000000000000243.
27. Ramanathan, K.; Antognini, D.; Combes, A.; Paden, M.; Zakhary, B.; Ogino, M.; MacLaren, G.; Brodie, D.; Sheker, K. Planning and provision of ECMO services for severe ARDS during the COVID-19 pandemic and other outbreaks of emerging infectious diseases. *Lancet Respir Med* **2020**, *8*, 518-526. doi: 10.1016/S2213-2600(20)30121-1.
28. Puślecki, Ł.; Dąbrowski, M.; Puślecki, M. Development of Innovation Cooperation in the Time of COVID-19 Pandemic. *European Research Studies Journal*, Special Issue 1, **2021**; 1049-1073. doi: 10.35808/ersj/2087.
29. Puślecki, Ł.; Czekajlo, M.; Puślecki, M. How Innovation Cooperation Supports the Improvement of Health Care in the CEE Region: The Case of ECMO for Greater Poland. European Research Studies Journal, Special Issue 1, **2021**; 1074-1095. doi: 10.35808/ersj/2088.
30. Smereka, J.; Puslecki, M.; Ruetzler, K.; Filipiak, K.J.; Jaguszewski, M.; Ladny, J.R.; Szarpak, L. Extracorporeal membrane oxygenation in COVID-19. *Cardiol J* **2020**, *27*, 216-217. doi: 10.5603/CJ.a2020.0053.
31. Smereka, J.; Szarpak, L.; Filipiak, K.J. Modern medicine in COVID-19 era. *Disaster Emerg Med J* **2020**, *5*, 103-105. doi: 10.5603/DEMJ.a2020.0012.
32. Sip, M.; Puslecki, M.; Dabrowski, M.; Klosiewicz, T.; Zalewski, R.; Ligowski, M.; Goszczynska, E.; Paprocki, C.; Grygier, M.; Lesiak, l. et al. Extended cardiopulmonary resuscitation: from high fidelity simulation scenario to the first clinical applications in Poznan out-of-hospital cardiac arrest program. *Perfusion* **2020** Dec 16:267659120981811. doi: 10.1177/0267659120981811.
33. Savarimuthu, S.; BinSaeid, J.; Harky, A. The role of ECMO in COVID-19: Can it provide rescue therapy in those who are critically ill? *J Card Surg* **2020**, *35*, 1298-1301. doi: 10.1111/jocs.14635.
34. Wiedemann, D.; Bernardi, M.H.; Distelmaier, K.; Goliasch, G.; Hengstenberg, C.; Hermann, A.; Holzer, M.; Hoetzenecker, K.; Klepetko, W.; Lang, G. et al. COVID-19 ECMO Arbeitsgruppe Medizinische Universität Wien. Empfehlungen für ECMO bei COVID-19-Patienten: Consensus Paper zur Extrakorporalen Membranoxygenierung der Medizinischen Universität Wien [Recommendations for Extracorporeal Membrane Oxygenation (ECMO) in COVID-19 Patients]. *Wien Klin Mag* **2020**, *10*, 1-6. doi: 10.1007/s00740-020-00349-x.
35. Shekar, K.; Badulak, J.; Peek, G.; Boeken, U.; Dalton, H.J.; Arora, L.; Zakhary, B.; Ramanathan, K.; Starr, J.; Akkanti, B. et al. Extracorporeal Life Support Organization COVID-19 Interim Guidelines: A consensus document from an international group of interdisciplinary Extracorporeal Membrane Oxygenation Providers. *ASAIO J* **2020**, *66*, 707-721. doi:10.1097/MAT.0000000000001193.
36. Broman, L.M.; Dirnberger, D.R.; Malfertheiner, M.V.; Aokage, T.; Morberg, P.; Naeshim, T.; Pappalardo, F.; Di Nardo, M.; Preston, T.;Burrell, A.J. et al. International Survey on Extracorporeal Membrane Oxygenation Transport. *ASAIO J* **2020**, *66*, 214-225. doi: 10.1097/MAT.0000000000000997.
37. Trąpczyński, P.; Puślecki, Ł.; Staszków, M. Determinants of Innovation Cooperation Performance: What Do We Know and What Should We Know? *Sustainability* **2018**, *10*, 1–32. http://doi.org/10.3390/su10124517

38. Puślecki, Ł. The development and management of alliance networks in the biopharmaceutical industry. W W. Sroka& Š. Hittmár, W. Sroka& Š. Hittmár (Red.), Management of Network Organizations: Theoretical Problems and the Dilemmas in Practice (ss. 199–213). *Springer International Publishing.* **2015**, http://doi.org/10.1007/978-3-319-17347-4_14
39. Puślecki, Ł.; Staszków, M. New Cooperation Modes: An Opportunity for Polish Biotechnological Clusters. *Managing Global Transitions: International Research Journal*, **2015**, *13*, 171–188.

List of tables and figures.

Table 1. Advantages of medical simulation and effective learning.

Table 2. Participant demographics, specialty and hospital settings with clinical practice and ECMO experience.

Table 3. Manual chest compression assessments with and without feedback device.

Table 4. Pre and post-course knowledge assessments (maximum score 15).

Figure 1. Pre and post-course cognitive self-assessments.

Figure 2. Pre and post-course behavioral self-assessments.

Figure 3. Pre and post-course technical self-assessments.

Figure 4. Pre and post-course knowledge assessment results.

Figure 5. Time to critical action completion in simulation scenario.

Figure 6. R&D Development: „know-how and knowledge transfer" in Education Center of Artificial Support in „ECMO for Greater Poland" Innovation Ecosystem.

Supplementary Materials:

Supplementary materials 1.

Supplementary materials 2.

Supplementary materials 3.

Click here to access/download;Figure;Figure1.docx

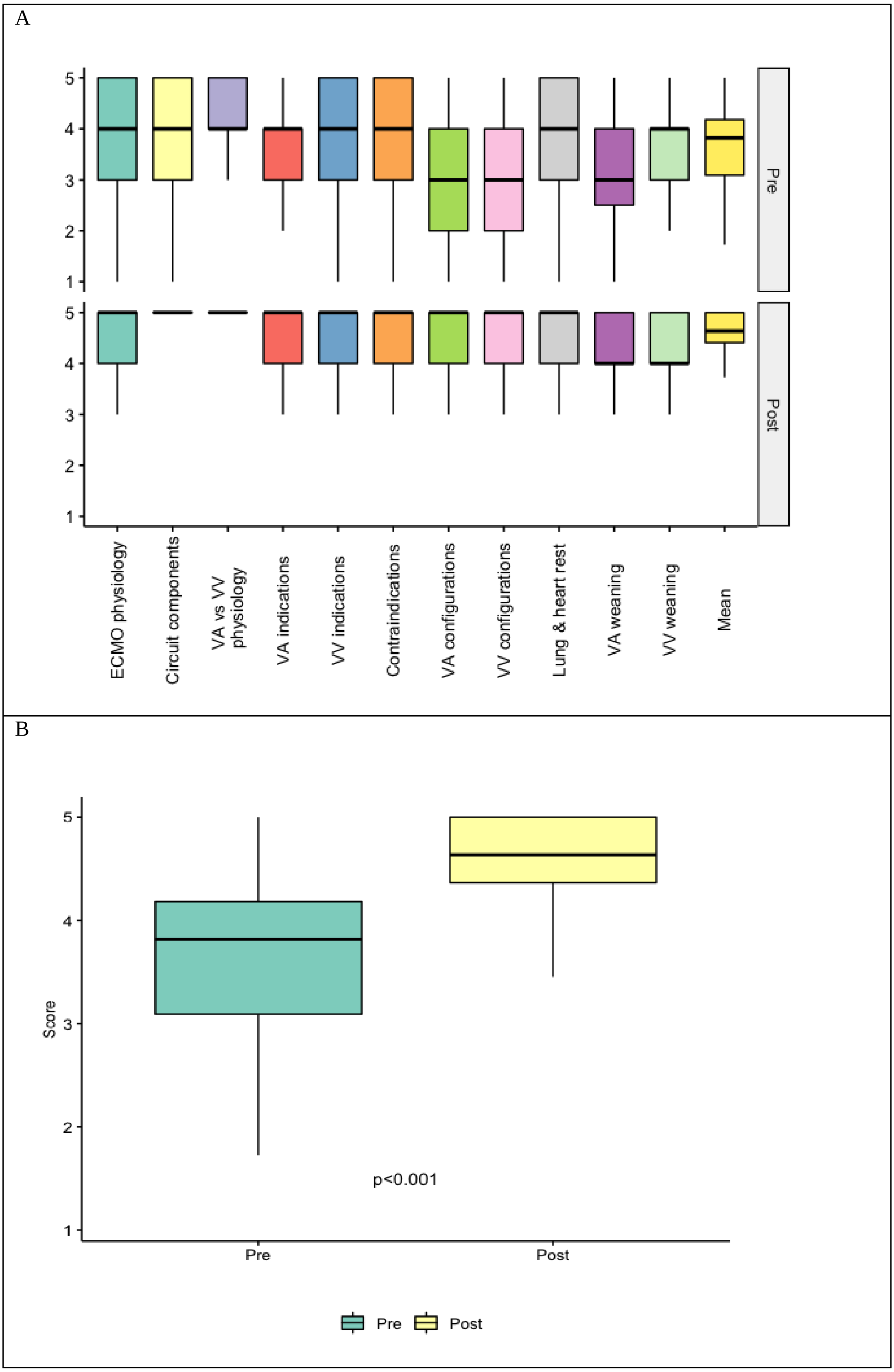

Figure 1. Pre and post-course cognitive self-assessments. A) Box and whisker plots of cognitive self-assessment scores by category. There was an overall increase in the median value for all assessed categories with less variability in the post-course period compared to the pre-course. B) Box and whisker plots of the mean cognitive self-assessment scores across all cognitive categories. There was a

statistically significant increase in the mean post-course scores compared to the pre-course period by Wilcoxon signed-rank test ($p<0.001$). ECMO = extracorporeal membrane oxygenation, VV = veno-venous, VA = veno-arterial.

Figure2

Click here to access/download;Figure;Figure2.docx

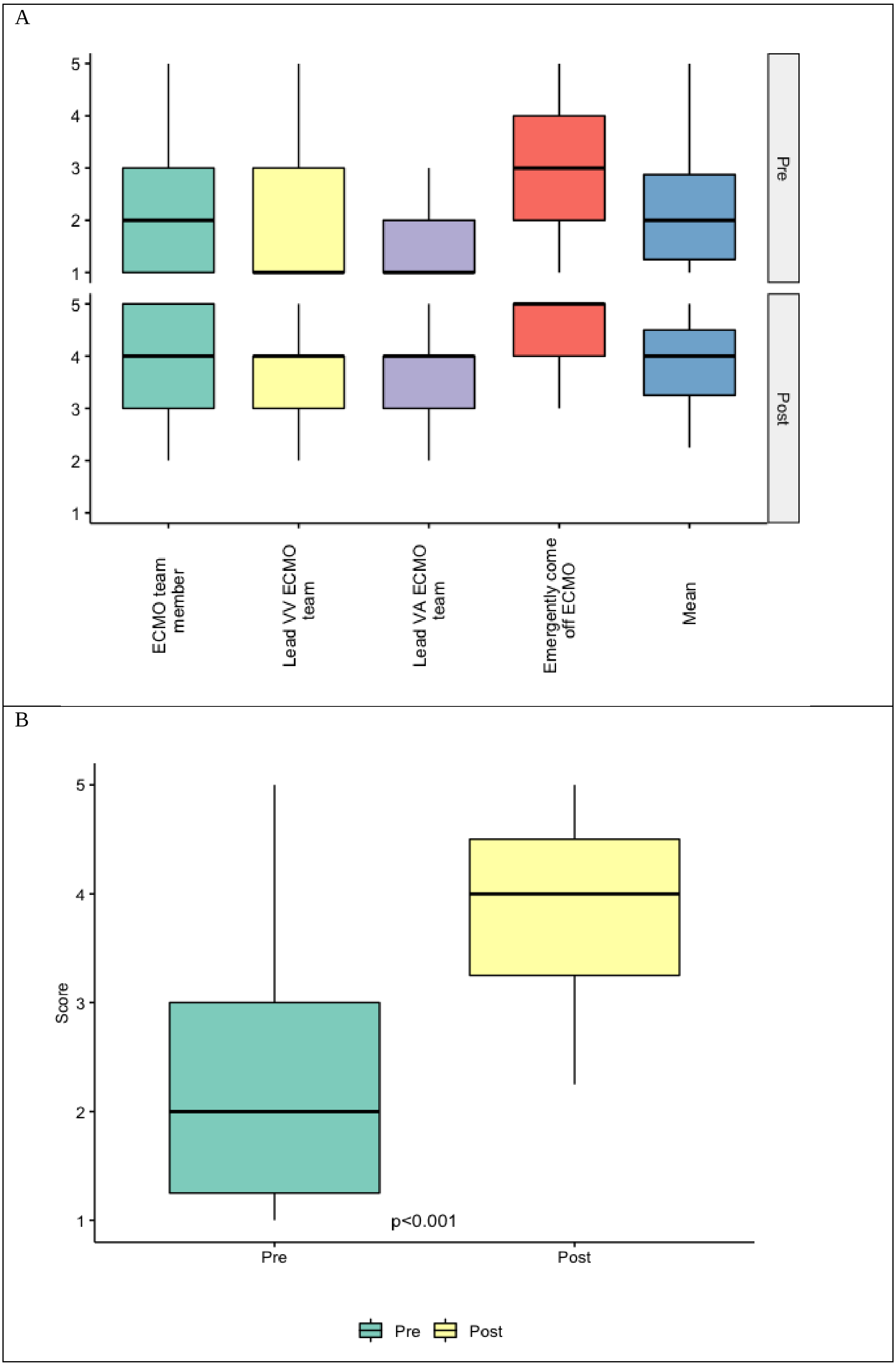

Figure 2. Pre and post-course behavioral self-assessments. A) Box and whisker plots of behavioral self-assessment scores by category. There was an overall increase in the median value for all assessed categories in the post-course period compared to the pre-course. B) Box and whisker plots of the mean behavioral self-assessment scores across all cognitive categories. There was a statistically significant increase in the mean post-course scores compared to the pre-course period by Wilcoxon signed-rank test ($p<0.001$). ECMO = extracorporeal membrane oxygenation, VV = veno-venous, VA = veno-arterial.

A

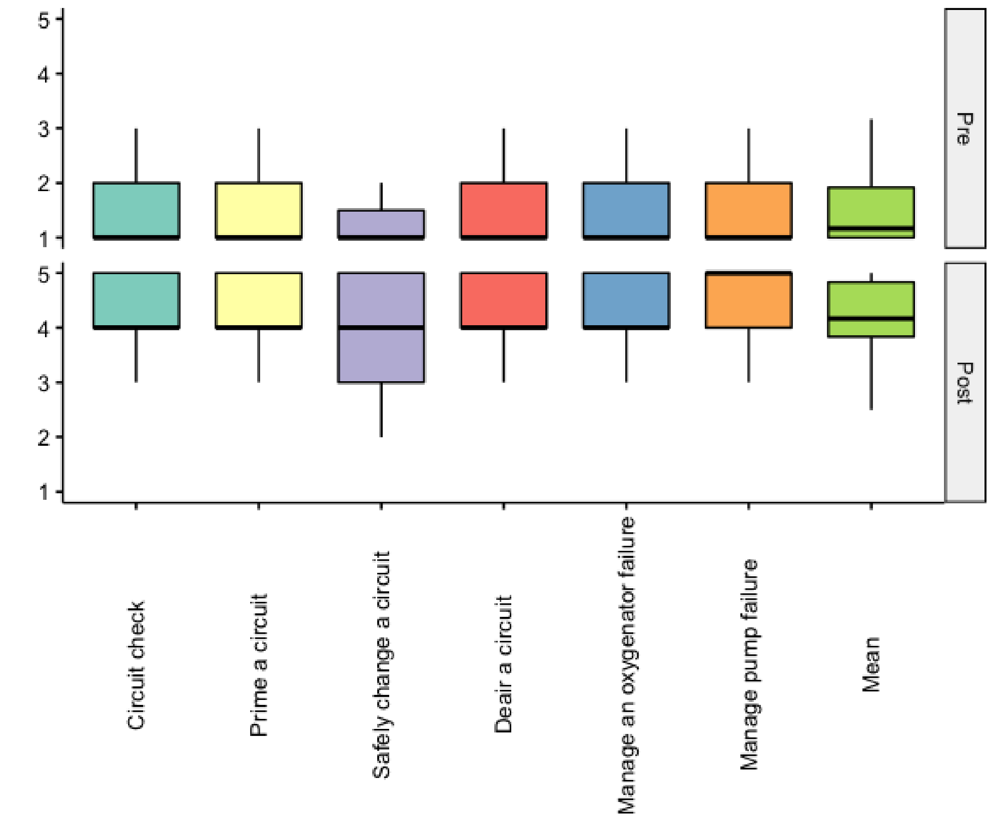

B

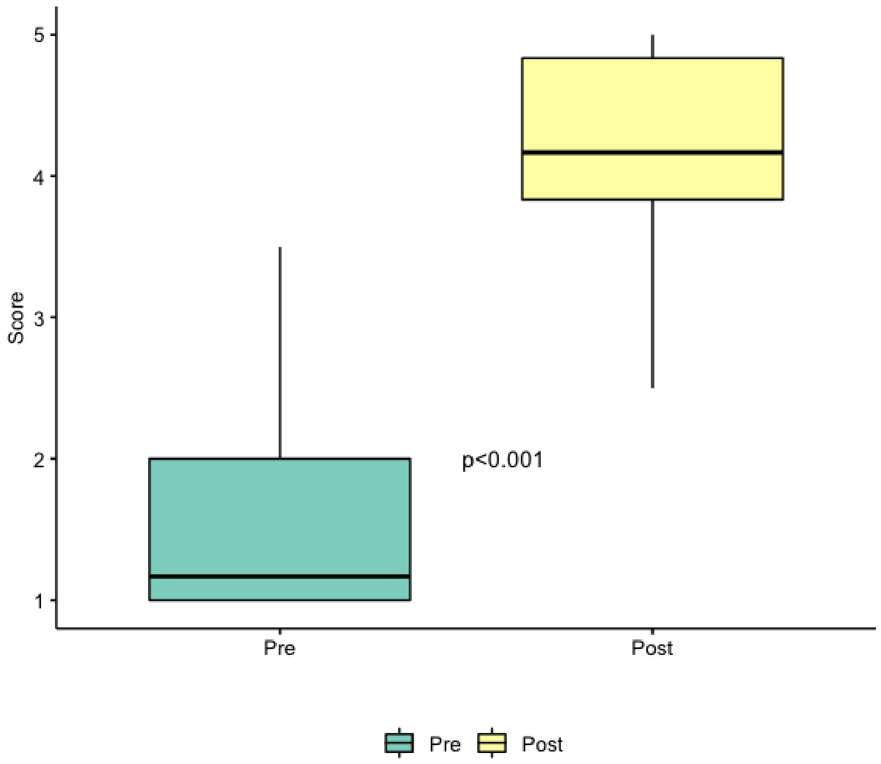

Figure 3. Pre and post-course technical self-assessments. A) Box and whisker plots of technical self-assessment scores by category. There was an overall increase in the median value for all assessed

categories in the post-course period compared to the pre-course. B) Box and whisker plots of the mean technical self-assessment scores across all cognitive categories. There was a statistically significant increase in the mean post-course scores compared to the pre-course period by Wilcoxon signed-rank test ($p<0.001$).

 Click here to access/download;Figure;Figure 4.docx

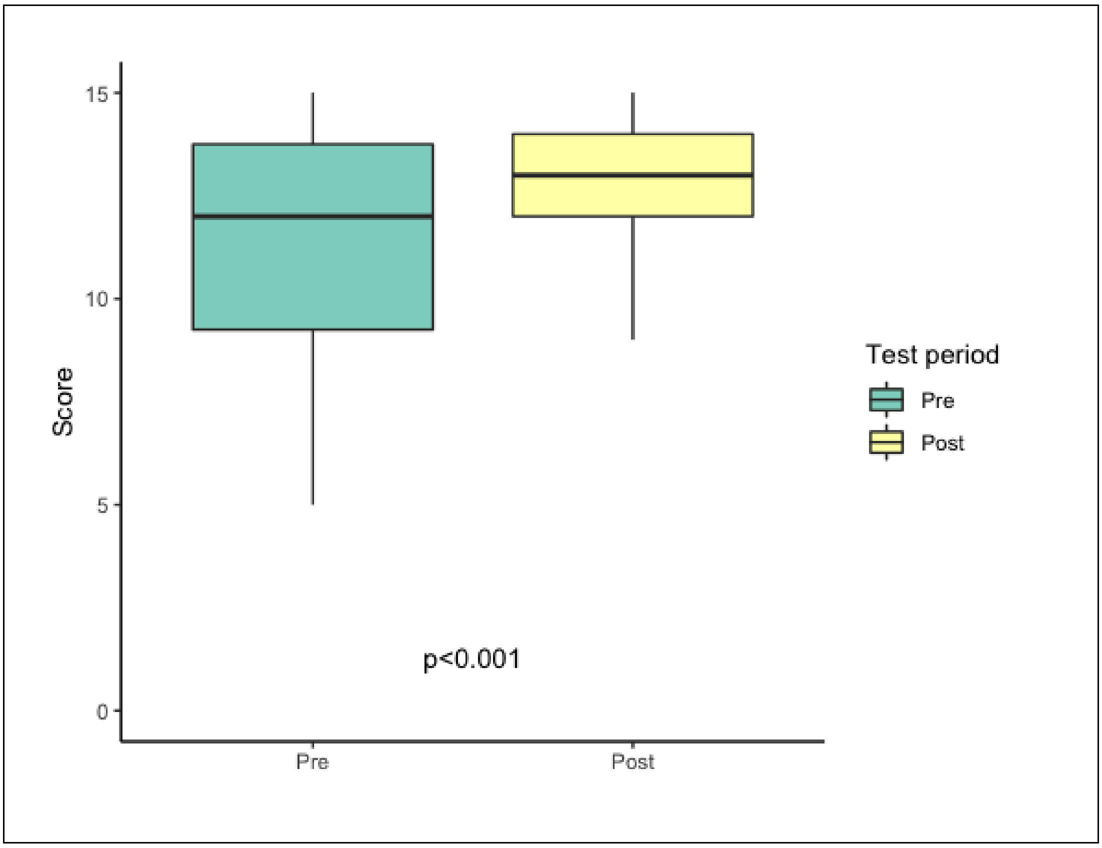

Figure 4. Pre and post-course knowledge assessment results. Box and whisker plots of the knowledge assessment results of the pre and post-course periods. There was a statistically significant increase in knowledge assessment scores in the post-course period (p<0.001) by Wilcoxon signed-rank test.

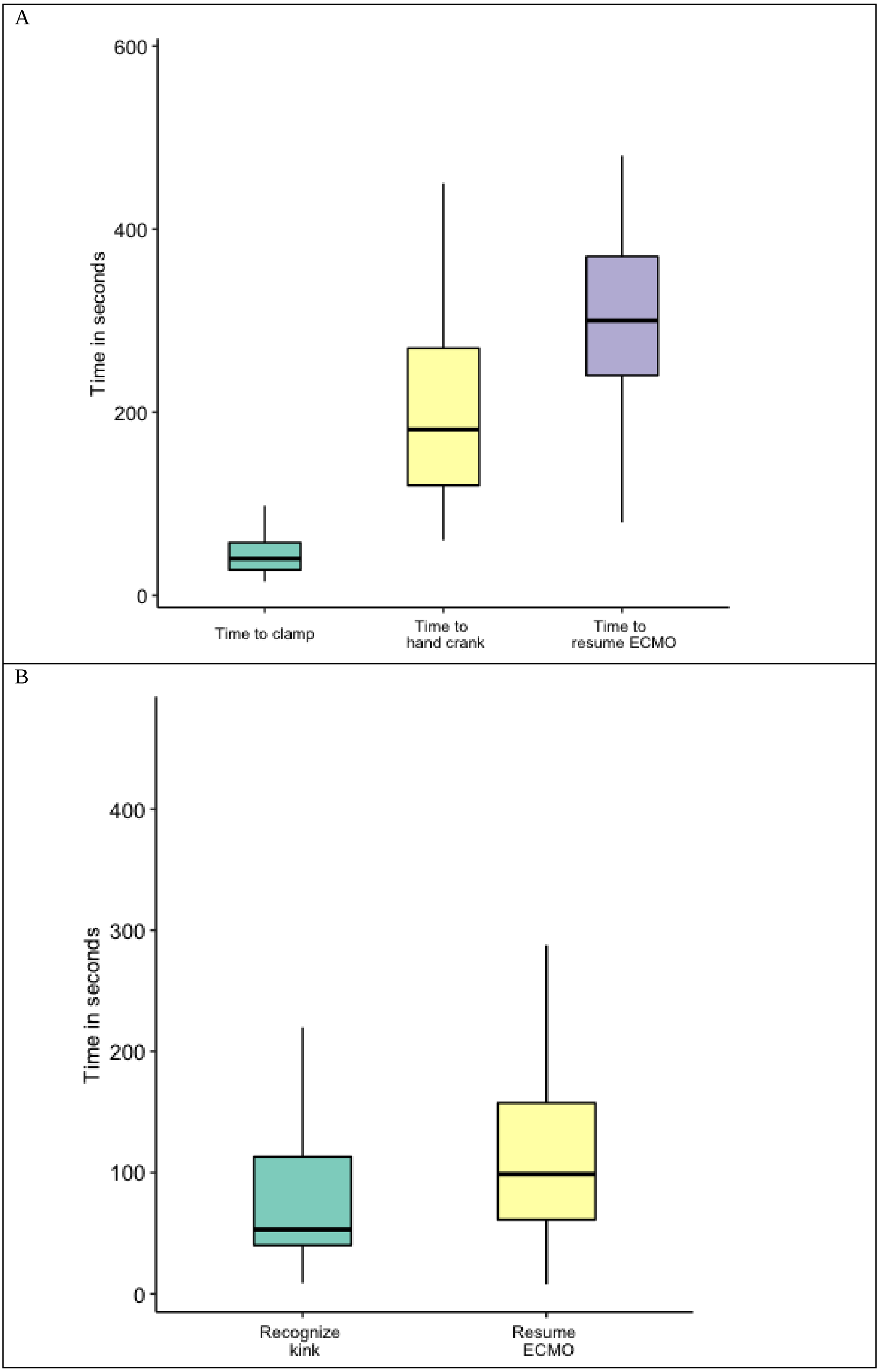

Figure 5. Time to critical action completion in simulation scenario. A) Box and whisker plot of time to completion of critical actions in the VA pump failure simulation scenario. B) Box and whisker plot of

time to completion of critical actions in the VV gas supply failure simulation scenario. VA = veno-arterial, VV = veno-venous.

Figure6 Click here to access/download;Figure;Figure 6.docx

Click here to access/download
Supporting Information
Supplementary material 1.docx

Click here to access/download
Supporting Information
Supplementary material 2.docx

Click here to access/download
Supporting Information
Supplementary material 3.docx
